# Two-Dimensional Metal–Organic Frameworks/Epoxy Composite Coatings with Superior O_2_/H_2_O Resistance for Anticorrosion Applications

**DOI:** 10.1021/acsami.4c04843

**Published:** 2024-07-12

**Authors:** Hao-Hsuan Hsia, You-Liang Chen, Yu-Ting Tai, Hong-Kang Tian, Chung-Wei Kung, Wei-Ren Liu

**Affiliations:** † Department of Chemical Engineering, R&D Center for Membrane Technology, Research Center for Circular Economy, Chung Yuan Christian University, Taoyuan 32023, Taiwan; ‡ Department of Graduate Institude of Applied Science and Technology, National Taiwan University of Science and Technology, Taipei City 106335, Taiwan; § Department of Chemical Engineering, National Cheng Kung University, Tainan City 70101, Taiwan; ∥ Program on Smart and Sustainable Manufacturing, Academy of Innovative Semiconductor and Sustainable Manufacturing, National Cheng Kung University, Tainan 70101, Taiwan; ⊥ Hierarchical Green-Energy Materials (Hi-GEM) Research Center, National Cheng Kung University, Tainan 70101, Taiwan

**Keywords:** hydrophobic, epoxy, anticorrosion, 2D structure, Zr-based MOFs, density functional theory

## Abstract

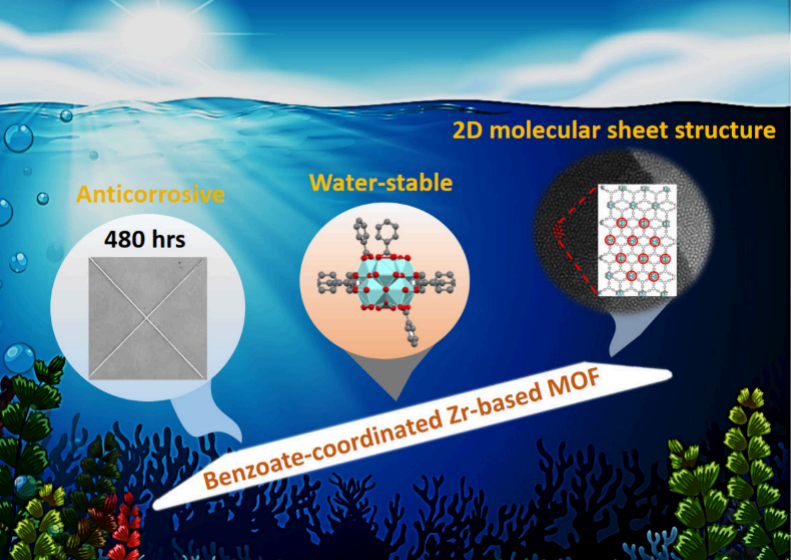

Corrosion protection technology plays a crucial role in preserving infrastructure, ensuring safety and reliability, and promoting long-term sustainability. In this study, we combined experiments and various analyses to investigate the mechanism of corrosion occurring on the epoxy-based anticorrosive coating containing the additive of two-dimensional (2D) and water-stable zirconium-based metal**–**organic frameworks (Zr-MOFs). By using benzoic acid as the modulator for the growth of the MOF, a 2D MOF constructed from hexazirconium clusters and BTB linkers (BTB = 1,3,5-tri(4-carboxyphenyl)benzene) with coordinated benzoate (BA-ZrBTB) can be synthesized. By coating the BA-ZrBTB/epoxy composite film (BA-ZrBTB/EP) on the surface of cold-rolled steel (CRS), we found the lowest coating roughness (RMS) of BA-ZrBTB/EP is 2.83 nm with the highest water contact angle as 99.8°, which represents the hydrophobic coating surface. Notably, the corrosion rate of the BA-ZrBTB/EP coating is 2.28 × 10^–3^ mpy, which is 4 orders of magnitude lower than that of the CRS substrate. Moreover, the energy barrier for oxygen diffusion through BA-ZrBTB/EP coating is larger than that for epoxy coating (EP), indicating improved oxygen resistance for adding 2D Zr-MOFs as the additive. These results underscore the high efficiency and potential of BA-ZrBTB as a highly promising agent for corrosion prevention in various commercial applications. Furthermore, this study represents the first instance of applying 2D Zr-MOF materials in anticorrosion applications, opening up new possibilities for advanced corrosion-resistant coatings.

## 1 Introduction

Corrosion, an inevitable natural process, results in material deterioration due to chemical or electrochemical reactions with the environment. It can weaken structural integrity, impair performance, and compromise safety across various domains, from industrial equipment and infrastructure to consumer goods, affecting economic and environmental sustainability.^1−5^ Metals, especially steel, are susceptible to corrosion, but cold-rolled steel (CRS) offers significant advantages in corrosion resistance. The smooth, scale-free surface of CRS reduces the likelihood of corrosion initiation and propagation, making it ideal for outdoor and marine use. Epoxy coatings are equally essential in corrosion protection and offer exceptional protection. The synergy of CRS and epoxy coatings is a robust strategy for safeguarding critical assets and infrastructure from corrosion.^6−10^

Metal–organic frameworks (MOFs) are emerging nanoporous materials with several unique features such as the regular and interconnected porosity, high specific surface area, and tunable chemical functional groups within the entire porous framework;^11,12^ such characteristics have thus motivated numerous researchers to utilize MOF-based materials in various applications including gas storage,^13,14^ separation,^15,16^ catalysis,^17−19^ sensors,^20−22^ and energy storage^23−25^ over the past two decades. Even though the poor chemical stability of most MOFs in water strongly restricts the practical usability of such highly porous materials,^26,27^ the development of group(IV) metal-based MOFs, such as zirconium-based MOFs (Zr-MOFs), has opened up opportunities for utilizing such materials in the applications that require exposure to humid environments or water.^27−29^ We thus reasoned that with the proper pore size and chemical functionality in the pore that can effectively retard the mass transfer of water and oxygen, Zr-MOFs could serve as the additive in the coating to further enhance the anticorrosion performance upon exposure to water. There are only a few published studies reporting the use of Zr-MOFs in anticorrosion coatings. The first example was reported by Ramezanzadeh and co-workers in 2021, who incorporated UiO-66, amino-functionalized UiO-66 (UiO-66-NH_2_), and UiO-66-NH_2_ modified with glycidyl methacrylate as the functional anticorrosive fillers in the epoxy coating.^30^ Bozorg et al. further developed the nanocomposite containing UiO-66-NH_2_ and carbon nanotubes as the additive in the epoxy coating for achieving the enhanced anticorrosion performance.^31^ Very recently, Zhu et al. synthesized the composite composed of UiO-66, mica nanosheets, 2-mercaptobenzothiazole, and polyethylenimine and utilized the resulting material in the epoxy-based anticorrosive coating.^32^ In the realm of anticorrosion materials, two-dimensional (2D) materials such as graphene, few-layer graphene, and graphene oxide have been widely utilized as additives because of their 2D structures with high aspect ratios that can provide exceptional gas-barrier properties^33−35^ and remarkable anticorrosion performances.^36^ We thus reasoned that by incorporating a highly water-stable Zr-MOF with a 2D structure and hydrophobic chemical functionality in its structure as an additive into the epoxy-based anticorrosive coating, the resulting performance should be better than that of the coatings with 3D Zr-MOFs such as UiO-66. But to date there is not any study reporting the use of 2D Zr-MOF in anticorrosion.

**Figure 1:**
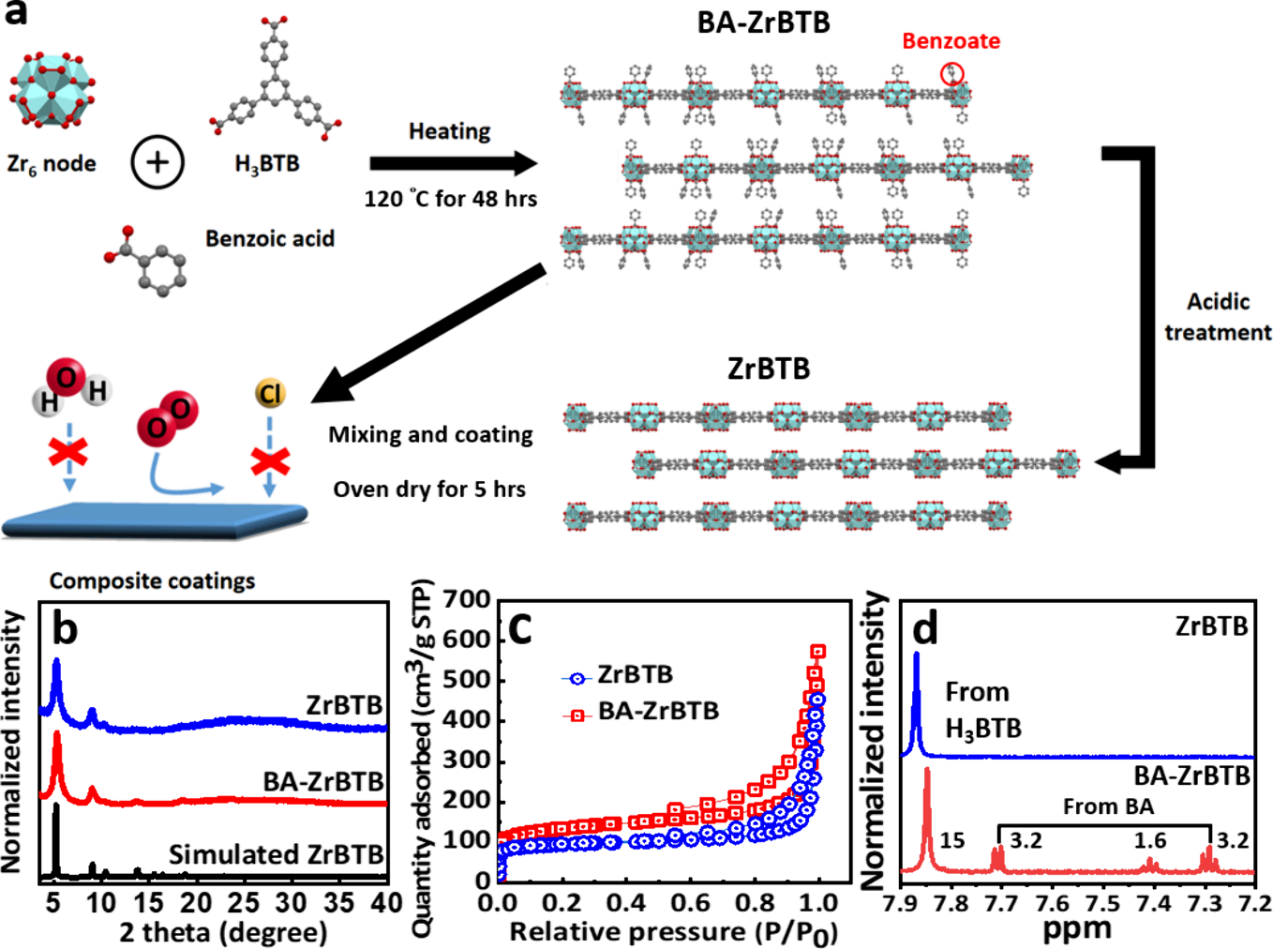
(a) Schematic diagram for the synthesis of ZrBTB and BA-ZrBTB and the incorporation of them into the epoxy-based anticorrosive coating. (b) XRD patterns and (c) nitrogen adsorption–desorption isotherms of ZrBTB and BA-ZrBTB. (d) NMR spectra of digested ZrBTB and BA-ZrBTB.

In this study, molecular sheets of a 2D Zr-MOF constructed from the hexazirconium clusters and BTB linkers, ZrBTB (BTB = 1,3,5-tri(4-carboxyphenyl)benzene),^37−39^ with and without the benzoate groups coordinated on its hexazirconium nodes,^40^ were synthesized and used as additives in the epoxy-based anticorrosive coatings (see Figure 1a). The results suggest that with the hydrophobic benzoate-functionalized ZrBTB (BA-ZrBTB) as the additive, the obtained anticorrosive coating can significantly outperform the coating without additives and those with the addition of ZrBTB, commercially available ZrO_2_, and H_3_BTB organic linker. Findings here provide insights into the potential use of 2D Zr-MOFs as effective additives in anticorrosion coatings aiming for various industrial applications.

## 2 Experimental Section

### 2.1 Chemicals

All chemicals including zirconium(IV) chloride anhydrous (ZrCl_4_, Acros Organics, 98%), 1,3,5-tri(4-carboxyphenyl)benzene (H_3_BTB, Alfa Aesar, 97%), benzoic acid (BA, Sigma-Aldrich, 99.5%), *N*,*N*-dimethylformamide (DMF, ECHO Chemical Co., Ltd., Taiwan, ≥99.8%), hydrochloric acid (HCl, J. T. Baker, 36.5–38.0%), dimethyl sulfoxide (DMSO, Duksan Pure Chemicals, 99%), acetone (ECHO Chemical Co., Ltd., Taiwan, 98%), dimethyl sulfoxide-*d*_6_ (DMSO-*d*_6_, Sigma-Aldrich, 99.9 atom % D), sulfuric acid-*d*_2_ solution (D_2_SO_4_, Sigma-Aldrich, 96–98 wt % in D_2_O, 99.5 atom % D), and potassium bromide (KBr, ≥99.0%, Honeywell Fluka) were used without purification. Deionized (DI) water was used as the water source throughout the whole work.

### 2.2 Synthesis of BA-ZrBTB and ZrBTB

ZrBTB powder was synthesized by following the procedure reported in our previous studies,^40,41^ with the use of benzoic acid as the modulator during the growth of MOF and the treatment in the HCl/DMSO mixture to fully remove the capping benzoate coordinated on the nodes. By following the synthetic procedure reported previously,^40^ the 2D MOF sample was also activated without performing the removal of coordinated benzoate groups, and the obtained material was named “BA-ZrBTB”.

### 2.3 Preparation of Composite Coatings

In the initial step, 1.0 wt % of Zr-MOF materials, specifically BA-ZrBTB and ZrBTB, was individually introduced into 7 mL of *N*,*N*-dimethylacetamide (DMAc, Macron, 99%) and subjected to ultrasonication for a duration of 30 min to ensure the formation of well-dispersed 2D Zr-MOFs suspensions. Next, 0.5 g of epoxy resin (EP; Sigma-Aldrich, 95.0%) was preheated to 50 °C for a period of 10 min to enhance its fluidity. Subsequently, the previously prepared suspension was combined with the EP and subjected to mechanical stirring for 2 h. To complete the formulation, 0.2 g of trimethylolpropane tris[poly(propylene glycol) and amine-terminated] ether (T-403; Sigma-Aldrich, 97.0%) was introduced as the curing resin into the mixture and stirred for a continuous 24 h duration, ensuring the achievement of uniform BA-ZrBTB/epoxy (BA-ZrBTB/EP) or ZrBTB/epoxy (ZrBTB/EP) slurries. Finally, the prepared slurry was applied onto the CRS substrate with a radius of 1.2 cm using a pipet (130 μL) and subjected to curing in an oven at 120 °C for 5 h. This process resulted in the formation of dried coatings with a thickness of 150 ± 7 μm.

### 2.4 Characterizations

The crystal structure and phase purity were determined using a powder X-ray diffractometer (PXRD, RIGAKU Ultima IV). The diffraction data were recorded in the range of 2θ = 3°–40°. The porosity of the samples can be determined by nitrogen adsorption–desorption isotherms, which were measured with an ASAP 2020 (Micromeritics). The ^1^H nuclear magnetic resonance (NMR) spectra of all samples were measured with a Bruker AVANCE 600 NMR spectrometer. The preparation of NMR samples was conducted as follows: 0.5 mg of the MOF solid was dissolved in a few drops of D_2_SO_4_ with 5 min of sonication. After that, 0.7 mL of DMSO-*d*_6_ was added to dilute the solution. The obtained solution was then sonicated for 20 min and filled into an NMR tube. The nanostructure and elemental distributions of composite materials and coatings were imaged by scanning electron microscopy (SEM, Hitachi SU8010) equipped with energy dispersive spectroscopy (EDS) and high-resolution transmission electron microscopy (HR-TEM, JEM-2100F, JEOL Ltd.) at an operating voltage of 200 kV. To prepare the sample for HR-TEM measurements, about 0.5 mg of MOF powder was fully dispersed in 1 mL of acetone, and the suspension was dropped onto a copper mesh. The prepared samples were then placed in a vacuum oven at 80 °C for evaporating the residual solvent. Contact angles were obtained on a FACE CBVP A3 where the vertical distance between the drop-casting position and the targeted substrate was consistently maintained and quantified using a standard ruler, measuring as approximately 1.6 cm. The chemical valence states of the elements in the as-prepared materials were investigated using X-ray photoelectron spectroscopy (XPS), which was performed on a Theta Probe (Thermo Scientific) equipped with a microfocused electron gun, a multiposition aluminum anode, and a monochromated X-ray source. All XPS spectra were corrected by referencing the C 1s peak to 284.8 eV. The Fourier transform infrared (FT-IR) absorption spectra were collected by using a Nicolet 6700 (Thermo Fisher Scientific) at room temperature with a scanning range of 4000–400 cm^–1^.

### 2.5 Electrochemical Characterization

Tafel plots and all electrochemical impedance spectroscopy (EIS) data of the corrosion-resistant steel (CRS), epoxy, and other composite coating films were conducted using an EC Lab (BioLogic potentiostat, model: SP-200). The CRS substrate utilized had a diameter of 18.2 mm and a thickness of 1.177 mm. The potential was scanned within the range of −0.25 to +0.25 V at a scan rate of 0.2 mV s^–1^ for Tafel plots. Before the electrochemical characterization, the working electrode was immersed in a sodium chloride (NaCl) solution at room temperature for two different time intervals: 30 min and 24 h. To ensure the reliability and statistical significance of the results, all raw data were collected through a minimum of five repetitions. Impedance measurements of the samples were carried out by performing EIS experiments across a frequency spectrum ranging from 1 MHz to 100 mHz. Oxygen permeation experiments were conducted using a gas permeability analyzer (GTR10-GPA) under a pressure of 1 atm (0.101 MPa) for a duration of 1 h. Salt spray tests were performed using an SST-A, following the standards outlined in ASTM B117, CNS 8886, and Z 8026. For these tests, carbon steel (CS) measuring 10 cm × 7 cm × 0.8 mm in dimensions was employed. Both CRS and CS materials were supplied by Shiny Chemical Industrial Co., Ltd.

### 2.6 Construction of ZrBTB and BA-ZrBTB Atomic Structures

The bulk atomic structure of ZrBTB is constructed with a space group of *P*3̅ based on experimental research.^41^ It comprises one atomic unit of Zr_6_(μ_3_-O)_4_(μ_3_-OH)_4_(BTB)_2_(OH)_6_(H_2_O)_6_ within a unit cell. The lattice parameters and atomic positions of ZrBTB are determined through powder X-ray diffraction data.^41^ It should be noted that experimental research did not reveal the positions and content of H atoms; hence, H atoms were manually added to the O atoms bonded to Zr centers to balance the overall charge. On both the top and bottom surfaces of the 2D ZrBTB, each side features three −OH groups and three −OH_2_ groups. For the BA-ZrBTB bulk structure, one of the −OH groups along with a neighboring −OH_2_ group on two adjacent Zr atoms in the Zr_6_O_8_ cluster is replaced by one benzoic acid molecule. Both the ZrBTB and BA-ZrBTB bulk structures were optimized using density functional theory (DFT) calculations.

For the surface (slab) structures of ZrBTB and BA-ZrBTB, we selected the (001) surface from the bulk due to its 2D characteristic nature. A vacuum layer of 20 Å was introduced between periodic slabs along the [001] direction to prevent interactions between slabs. This methodology aligns with similar single-layer atomic models employed in previous computational investigations. Our experimental data, as shown in Figure 1d, indicate that the presence of BA molecules within each Zr_6_O_8_ cluster ranges from 1 to 2. To emulate this scenario while optimizing the computational efficiency, we positioned a single BA molecule on the surface of ZrBTB, thus forming the simplified BA-ZrBTB structure. This approach not only captures the influence of BA but also mitigates computational expenses. To identify potential adsorption sites for O_2_ and H_2_O molecules on the surfaces of both MOFs, we initially employed the Delaunay triangulation algorithm,^42^ implemented in the adsorption module of Pymatgen,^43^ an open-source Python package. Given that the slab model includes over 150 atoms, exploring the numerous possible orientations of the O_2_ and H_2_O molecules on the surface could be computationally demanding and costly. Consequently, we utilized a universal machine-learning interatomic potential, the Crystal Hamiltonian Graph Neural Network (CHGNet),^44^ which was fine-tuned using our DFT calculations that included O_2_/H_2_O on the surfaces of ZrBTB and BA-ZrBTB. This approach enabled us to efficiently screen for the most favorable orientations of O_2_ and H_2_O molecules from a total of 1870 different possibilities, based on their energies computed via CHGNet. We selected the six structures with the lowest energy for more accurate DFT calculations to analyze their adsorption energies.

### 2.7 DFT Calculations

In this research, the atomic structures were analyzed using DFT via the Vienna Ab initio Simulation Package (VASP),^45^ utilizing the Perdew–Burke–Ernzerhof (PBE) version of the generalized gradient approximation (GGA)^46^ and the projector-augmented wave (PAW) technique.^47^ The configurations of valence electrons for the elements included were 4s^2^5s^1^4p^6^4d^3^ for Zr, 1s^1^ for H, 2s^2^2p^2^ for C, and 2s^2^2p^4^ for O. Optimization of both atom positions and lattice parameters was carried out for bulk structures to remove internal stress, whereas for surface structures, only atom positions were adjusted. The process of geometry optimization utilized a 500 eV cutoff energy and the Gaussian smearing approach for orbital occupancies with a smearing width of 0.1 eV. The criteria for convergence in the electronic and ionic relaxation phases were an energy discrepancy of less than 10^–5^ eV and a force below 0.03 eV Å^–1^, respectively. A *k*-spacing of 0.03·2π/Å in the reciprocal space as per the Monkhorst–Pack grid^48^ was chosen to ensure a total energy shift with *k*-grids under 1 meV/atom.

The adsorption energy, *E*_ad_, of O_2_ and H_2_O molecules on ZrBTB and BA-ZrBTB surfaces is calculated by following equation:
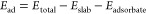
where *E*_total_, *E*_slab_, and *E*_adsorbate_ are the energy of final absorbed structure, pure slab, and isolated O_2_ and H_2_O molecules, respectively.

## 3 Results and Discussion

**Figure 2:**
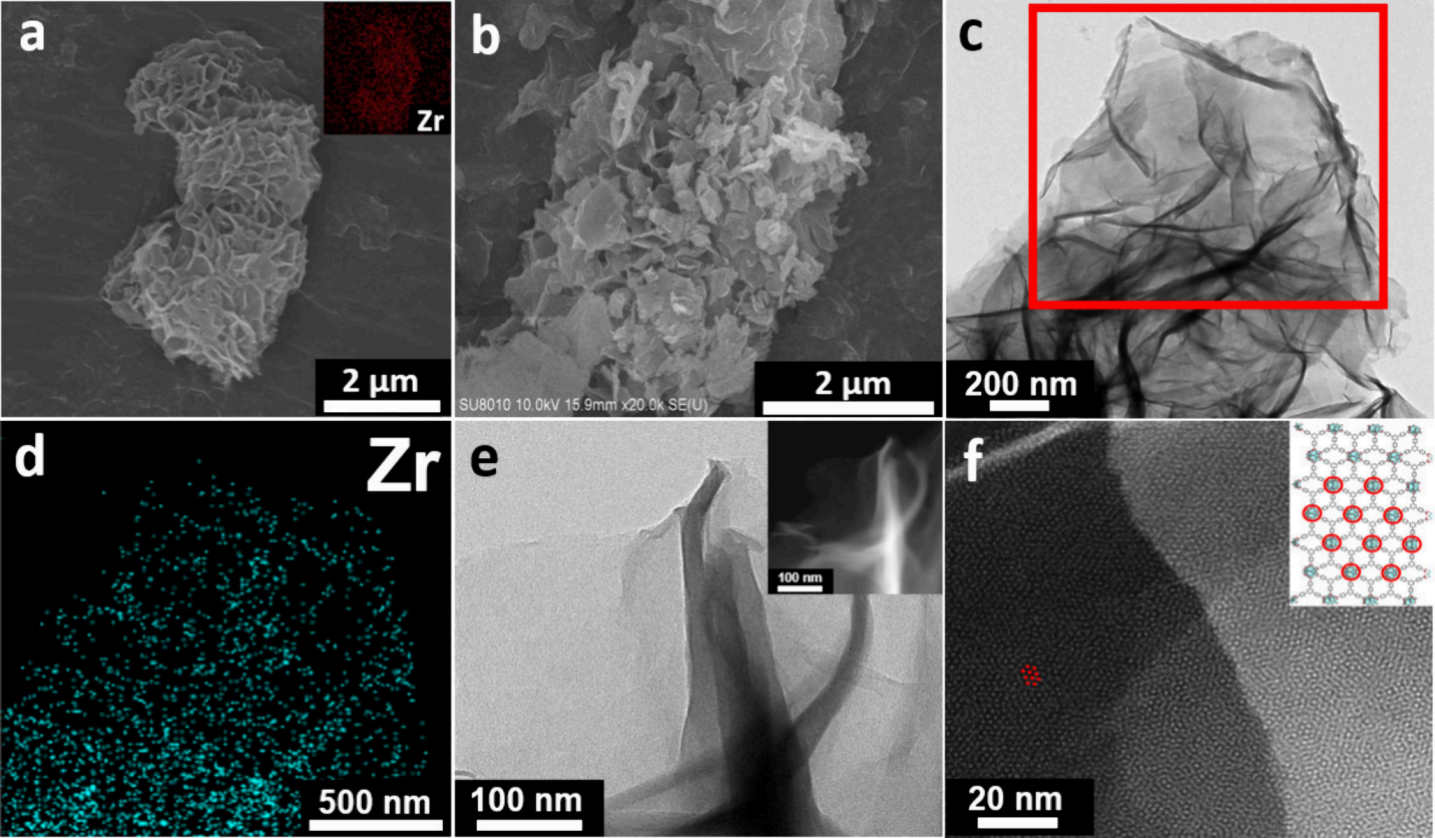
(a) SEM images at a low magnification with an inset showing the EDS elemental mapping for Zr and (b) high-magnification SEM image of BA-ZrBTB. (c) Low-magnification TEM image, (d) EDS elemental mapping for Zr collected from the rectangular region shown in (c), and (e) HR-TEM image of the BA-ZrBTB nanosheet. Dark-field HR-TEM image of BA-ZrBTB is shown in the inset of (e). (f) HAADF-STEM image of the single BA-ZrBTB sheet showing lattice fringes of the 2D MOF. Crystalline structure of ZrBTB with one layer is shown in the inset of (f).

Figure 1a shows the synthetic process of the hydrophobic and anticorrosive BA-ZrBTB/epoxy (BA-ZrBTB/EP) composite coatings. The water-stable BA-ZrBTB consists of BTB linker, Zr_6_ cluster, and the benzoic acid as modulator. In addition, the BA-ZrBTB was subjected to acid treatment to remove the coordinated benzoic acid for tailoring the properties of material without the change of whole MOF structure, which was named ZrBTB. Subsequently, these two powders were incorporated as additives during the synthesis of the composite coatings for further applications. PXRD patterns of BA-ZrBTB, ZrBTB, and simulated ZrBTB are shown in Figure 1b. The diffraction peaks located at 2θ = 5.2°, 8.9°, and 10.4° can be observed in the patterns of all the MOF-based samples, which correspond to the (100), (110), and (200) crystal planes of ZrBTB, respectively. This observation suggests that the BA-ZrBTB and ZrBTB were successfully synthesized with high crystallinity.^49^ Moreover, XRD patterns of ZrO_2_^50,51^ and H_3_BTB are included in Figure S1. The diffraction peaks of H_3_BTB cannot be observed in the samples of BA-ZrBTB and ZrBTB, suggesting that the residual H_3_BTB linker has been predominantly eliminated during the synthetic process of the MOF. Nitrogen adsorption–desorption isotherms of BA-ZrBTB and ZrBTB are shown in Figure 1c to investigate their porosities. The isotherms exhibit a sharp N_2_ uptake at low relative pressures and a hysteresis loop in the region of high relative pressure, which correspond to the microporosity of the samples and the interspace between stacked MOF particles, respectively. In addition, BA-ZrBTB and ZrBTB possess the Brunauer–Emmett–Teller (BET) surface areas of 490 and 310 m^2^ g^–1^, respectively; these findings agree well with the characteristics of BA-ZrBTB and ZrBTB.^52,53^^1^H NMR spectra of digested MOF powders were used to confirm the amount of benzoate grafting on each hexazirconium cluster of BA-ZrBTB and the removal of benzoate after the acidic treatment of BA-ZrBTB. As shown in Figure 1d, three sets of peaks located at 7.3, 7.4, and 7.7 ppm originating from benzoic acid, and a peak at 7.85 ppm corresponding to 15 protons of the H_3_BTB linker,^54^ can be observed in the ^1^H NMR spectrum of digested BA-ZrBTB. However, no signals from benzoic acid can be observed in the spectrum of digested ZrBTB, verifying the successful removal of benzoate after the acidic treatment. According to the integrated peak areas shown in Figure 1d, there are on average 1.6 benzoate ligands per BTB linker, namely, 3.2 benzoate ligands coordinated on each Zr_6_ node in BA-ZrBTB, which agrees well with the loading reported in the literature.^54^ SEM images of BA-ZrBTB collected at low and high magnifications are shown in Figures 2a and 2b, respectively. The flower-like morphology composed of ultrathin 2D nanosheets can be observed in these images, which is consistent with the reported morphology of ZrBTB.^54^ The EDS mapping signals of Zr are displayed in the inset of Figure 2a, verifying the uniform spatial distribution of the Zr_6_ clusters in the sample. From the TEM image of BA-ZrBTB, the nanosheets of BA-ZrBTB can be clearly obeserved (Figure 2c), and Zr signals collected by EDS are evenly distributed on the 2D layers, as shown in Figure 2d. In addition, the HR-TEM and corresponding HAADF-STEM images are shown in Figures 2e and 2f, respectively. Bright spots arranging regularly in a pattern of the *kgd* topology can be clearly seen on the 2D sheet, which indicates the successful synthesis of the 2D Zr-MOF; also see the crystalline structure of ZrBTB with *kgd* topology displayed in the inset of Figure 2f. SEM, TEM, EDS mapping, and HAADF-STEM experiments were also conducted for ZrBTB, and as shown in Figure S2, the similar 2D morphology, uniform spatial distribution of zirconium, and the *kgd* topology can also be observed. On the other hand, SEM and TEM images of both BA-ZrBTB and Zr-BTB were also collected and are presented as Figure S3, which reveals that there is not any morphological change after the removal of benzoate ligands from the 2D MOF.

**Figure 3:**
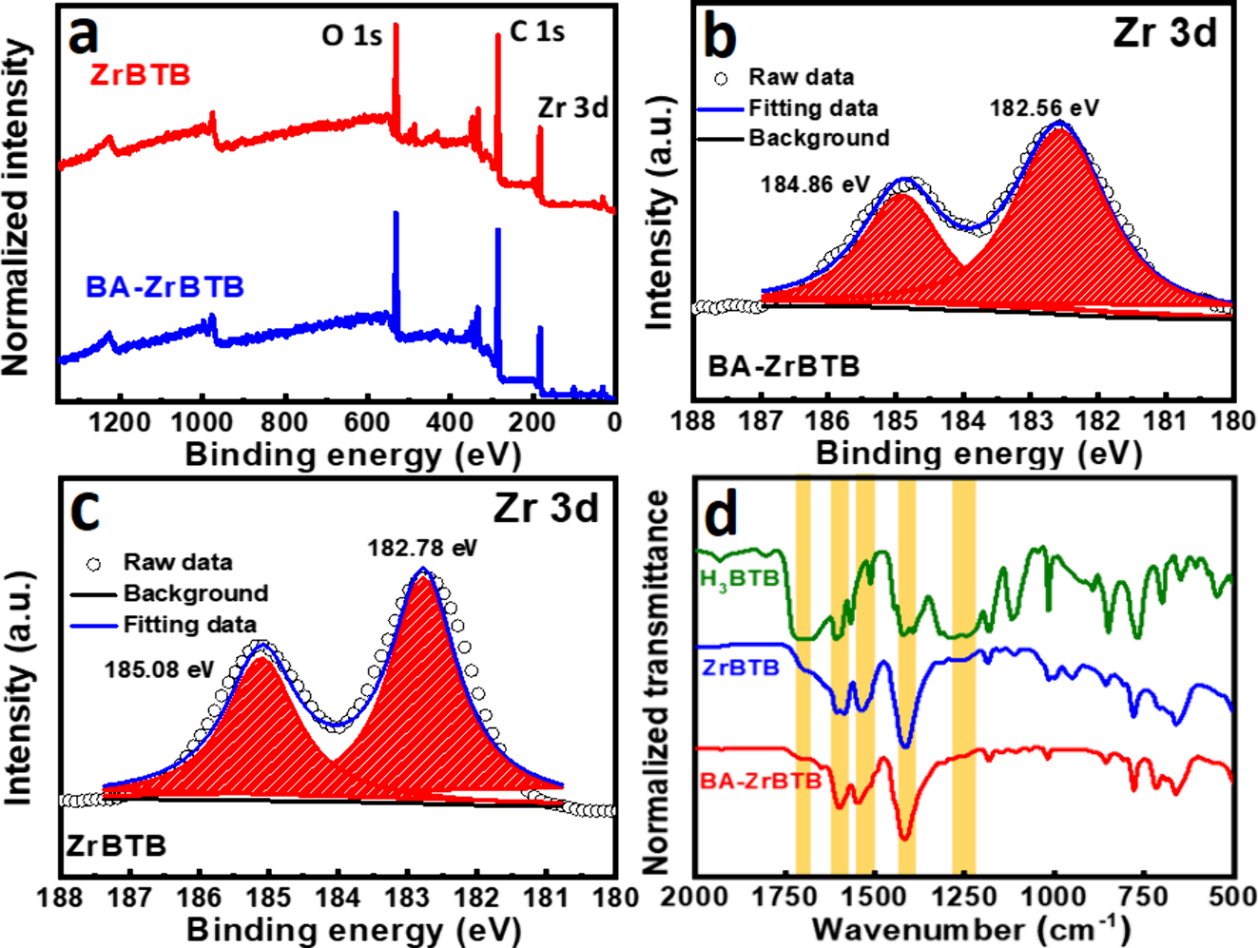
(a) XPS survey spectra of ZrBTB and BA-ZrBTB. (b, c) Zr 3d spectrum of BA-ZrBTB and ZrBTB. (d) FT-IR spectra of H_3_BTB, ZrBTB, and BA-ZrBTB.

Survey XPS spectra of ZrBTB and BA-ZrBTB are shown in Figure 3a, exhibiting the peaks of C 1s, O 1s, and Zr 3d in both spectra. The Zr 3d spectra of ZrBTB and BA-ZrBTB can be deconvoluted into two peaks. As shown in Figures 3b and 3c, the Zr signals of BA-ZrBTB exhibit a negative shift of 0.2 eV in binding energy compared to those of ZrBTB, verifying the successful coordination of benzoate on the Zr_6_ nodes; similar results were also observed in the literature.^54^ FTIR spectra of ZrBTB, BA-ZrBTB, and the H_3_BTB linker were further collected. As shown in Figure 3d, three peaks at 1610, 1543, and 1413 cm^–1^ can be observed in spectra of both ZrBTB and BA-ZrBTB, confirming the presence of O–C–O and aromatic C=C bonds in both MOF-based samples.^55^ In addition, two strong peaks centered at 1704 and 1252 cm^–1^, which correspond to the C=O vibrations and C–OH bending of the free carboxylic acid, respectively, can be observed in the spectrum of H_3_BTB. These signals become much weaker in the spectra of BA-ZrBTB and ZrBTB, indicating that the presence of free uncoordinated H_3_BTB linkers in both BA-ZrBTB and ZrBTB is negligible.

**Figure 4:**
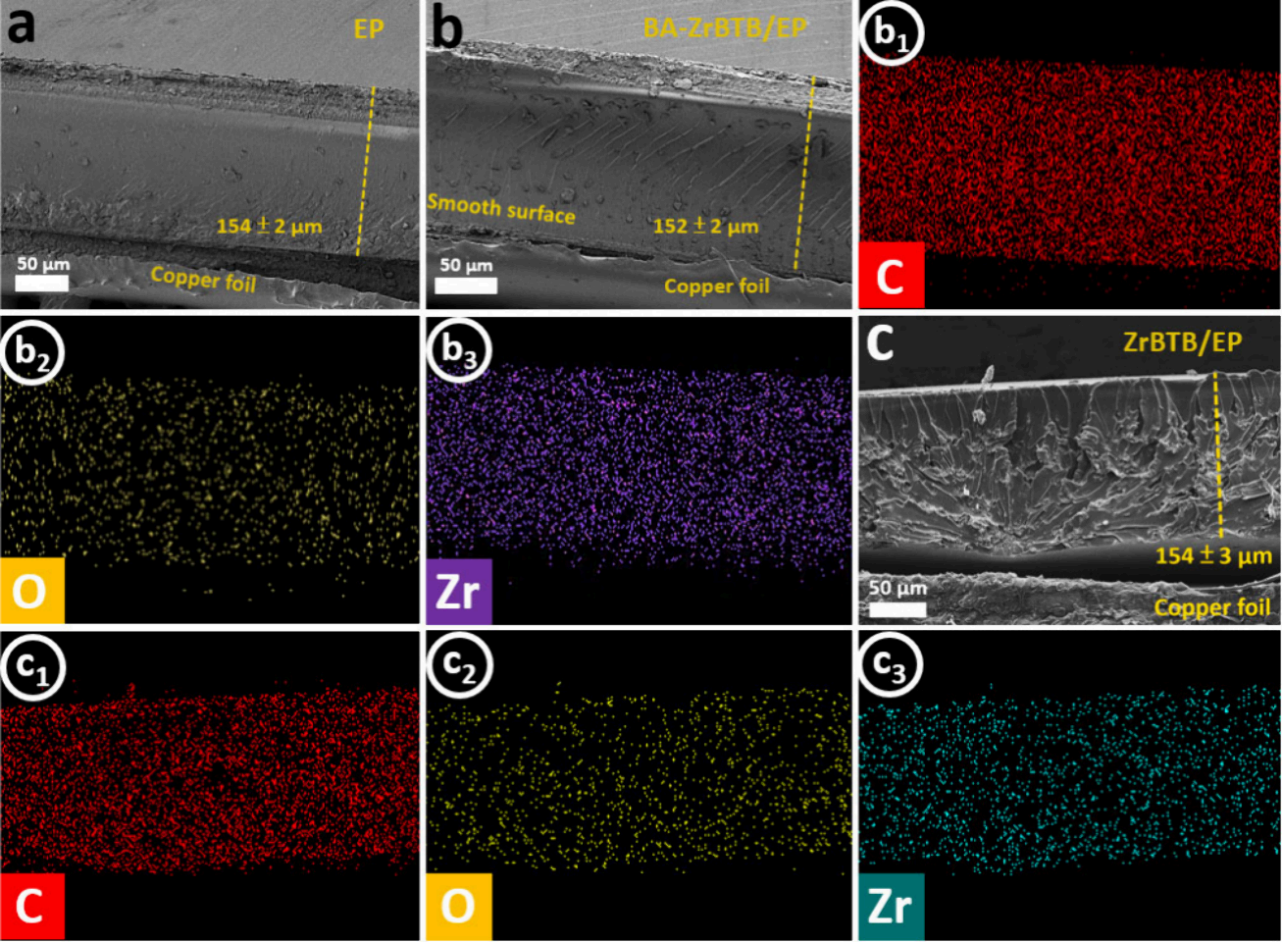
High-resolution cross-sectional SEM images of (a) the EP coating, (b) the BA-ZrBTB/EP composite coating, and (c) the ZrBTB/EP composite coating, including additional elemental mapping for C (b1, c1), O (b2, c2), and Zr (b3, c3).

To investigate the morphology and elemental distribution within the coatings, cross-sectional SEM experiments and EDS mapping analysis were conducted on the EP, BA-ZrBTB/EP, and ZrBTB/EP coatings, as depicted in Figure 4. These images reveal coating thicknesses of approximately 150 μm. Upon closer examination, the EP coating shown in Figure 4a exhibited a smooth, uniform, and flat surface, indicative of a cohesive and well-adhered structure. This structural integrity is essential for the successful creation of compact and robust coatings. However, it is worth noting the presence of particle-like structures distributed on the cross-sectional surface of the EP coating, likely a result of the coating breaking process during the preparation of the SEM cross-section sample. Furthermore, the analysis of BA-ZrBTB/EP in Figure 4b reveals the presence of more particles on the surface compared with the EP coating. This can be associated with the addition of 2D BA-ZrBTB powder. Nevertheless, the coating surface maintains its smooth texture, indicating that the incorporation of 2D BA-ZrBTB powder does not significantly alter the overall morphology of the coating. Furthermore, the SEM analysis reveals an orderly arrangement of surface morphology. In comparison to the flawless surface of the EP coating, the BA-ZrBTB/EP coating exhibits a more organized arrangement, evident in the top-right to bottom-left and top to bottom directions. However, the same arrangement is not observed in the ZrBTB/EP coating, as shown in Figure 4c. This difference may be attributed to the random breaking of the coating, even though all coating samples underwent the same breaking process. These variations in surface morphology could have implications for the coating performance, particularly in terms of the anticorrosion and hydrophobic properties. The smooth surface of the coatings can enhance surface interactions and barrier properties, potentially leading to better anticorrosion performance compared to coatings with rougher surfaces.

To ensure the uniform distribution of elements within the coatings, EDS mapping was employed. In the case of the BA-ZrBTB/EP coating, the bright dots representing carbon (C), oxygen (O), and zirconium (Zr) are evenly distributed throughout the entire cross-sectional coating surface. Similarly, uniform spatial distributions of all elements can also be observed in the cross-sectional SEM image of the ZrBTB/EP coating. These uniform distributions of elements not only indicate the remarkable dispersion of elements within the coating but also affirm the reliability of the subsequent electrochemical tests and results of the coatings.

**Figure 5:**
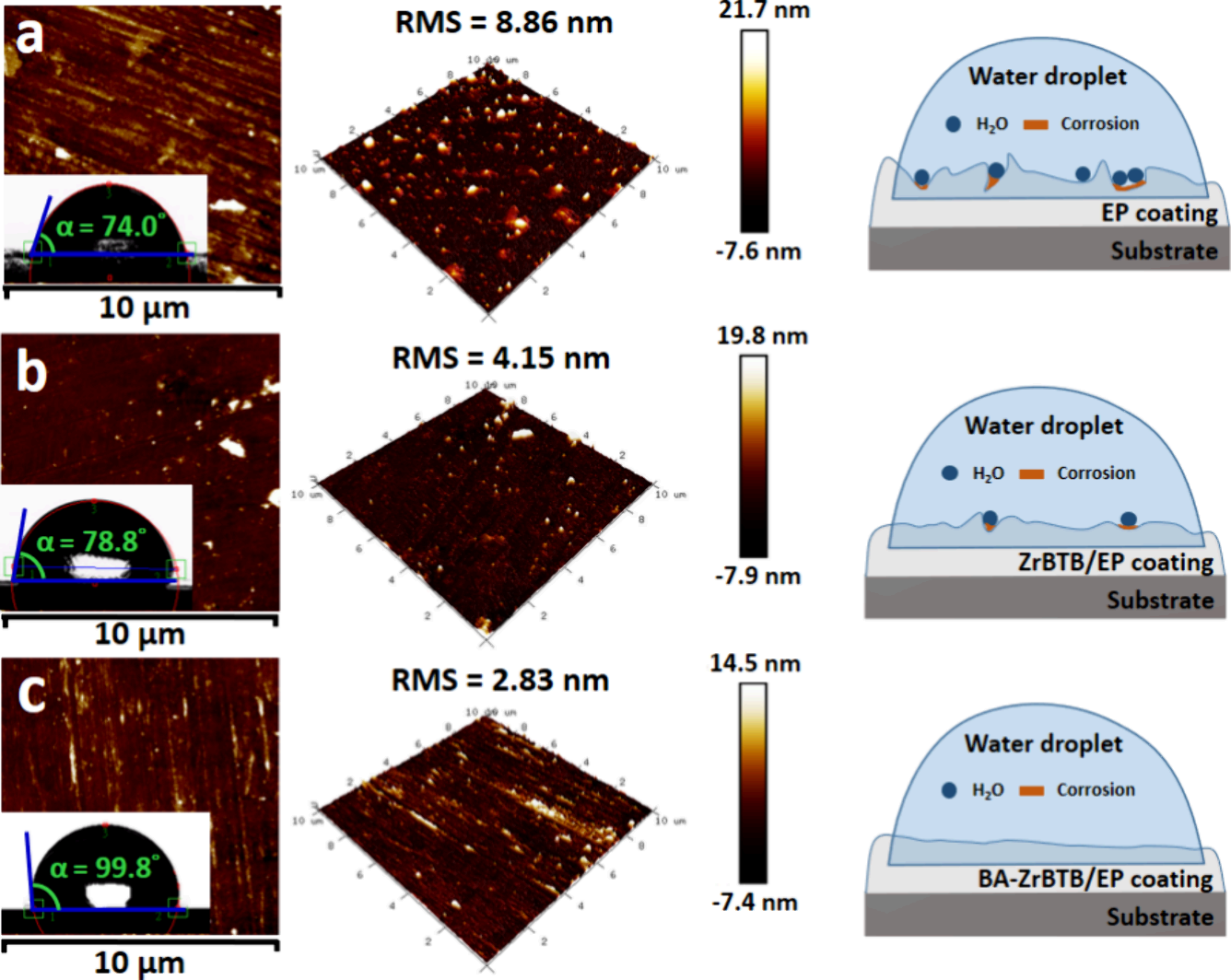
Three-dimensional AFM topography images along with roughness (RMS) analysis, with water contact angle (WCA) data shown as inset images and schematic diagrams regarding the effect of roughness on the corrosion process for (a) EP, (b) ZrBTB/EP, and (c) BA-ZrBTB/EP coatings.

3D AFM analysis was employed to determine the thickness and roughness of the coating layers. Figures 5a, 5b, and 5c display the AFM data of EP, ZrBTB/EP, and BA-ZrBTB/EP coatings on copper (Cu), respectively, with the corresponding data for water contact angle as the inset images and the schematic diagrams shown in the right panels. In these images, the dark areas indicate regions with lower coating thickness value, while the bulged regions represent areas with higher thickness value. As previously observed in the SEM cross-sectional images, the EP coating exhibits a smooth surface with a surface roughness (RMS) of 8.86 nm. Following the addition of 2D BA-ZrBTB into the coating, the RMS value significantly decreases to 2.83 nm. This decrease reflects the flat and flawless surface of the coating. However, the RMS value for ZrBTB/EP was tested as 4.15 nm, which was lower than that of EP but higher than the BA-ZrBTB/EP coating. The lowest RMS value observed for BA-ZrBTB/EP may be attributed to the better compatibility of the additive, solvent, and epoxy resin, potentially enhancing the anticorrosive ability to some extent. To assess the hydrophobicity of the coating surface, water contact angle (WCA) analysis was employed. The hydrophobic angles for the EP, BA-ZrBTB/EP, and ZrBTB/EP coatings are 74.0°, 99.8°, and 78.8°, respectively, following the same trend as that of RMS values. The higher hydrophobicity of the BA-ZrBTB/EP coating should be attributed to the benzoate groups coordinated on the nodes of BA-ZrBTB, which are more hydrophobic compared with the terminal −OH/–OH_2_ pairs present on the nodes of ZrBTB. BA-ZrBTB/EP exhibited the lowest RMS value and the highest WCA, simultaneously, indicating the highest likelihood of superior anticorrosion performance due to its flat and hydrophobic coating surface. The mechanism of how coating roughness affected the corrosion reaction is illustrated in the schematic diagram in Figure 5. When the coating surface was rough, water may be trapped in the defects with a lower height, resulting in a higher corrosion rate at specific points on the coating. This can ultimately lead to deterioration of the protective coating layer and corrosion of the substrate. Therefore, the control of coating roughness was a crucial factor in ensuring high anticorrosion performance.^56−58^

**Figure 6:**
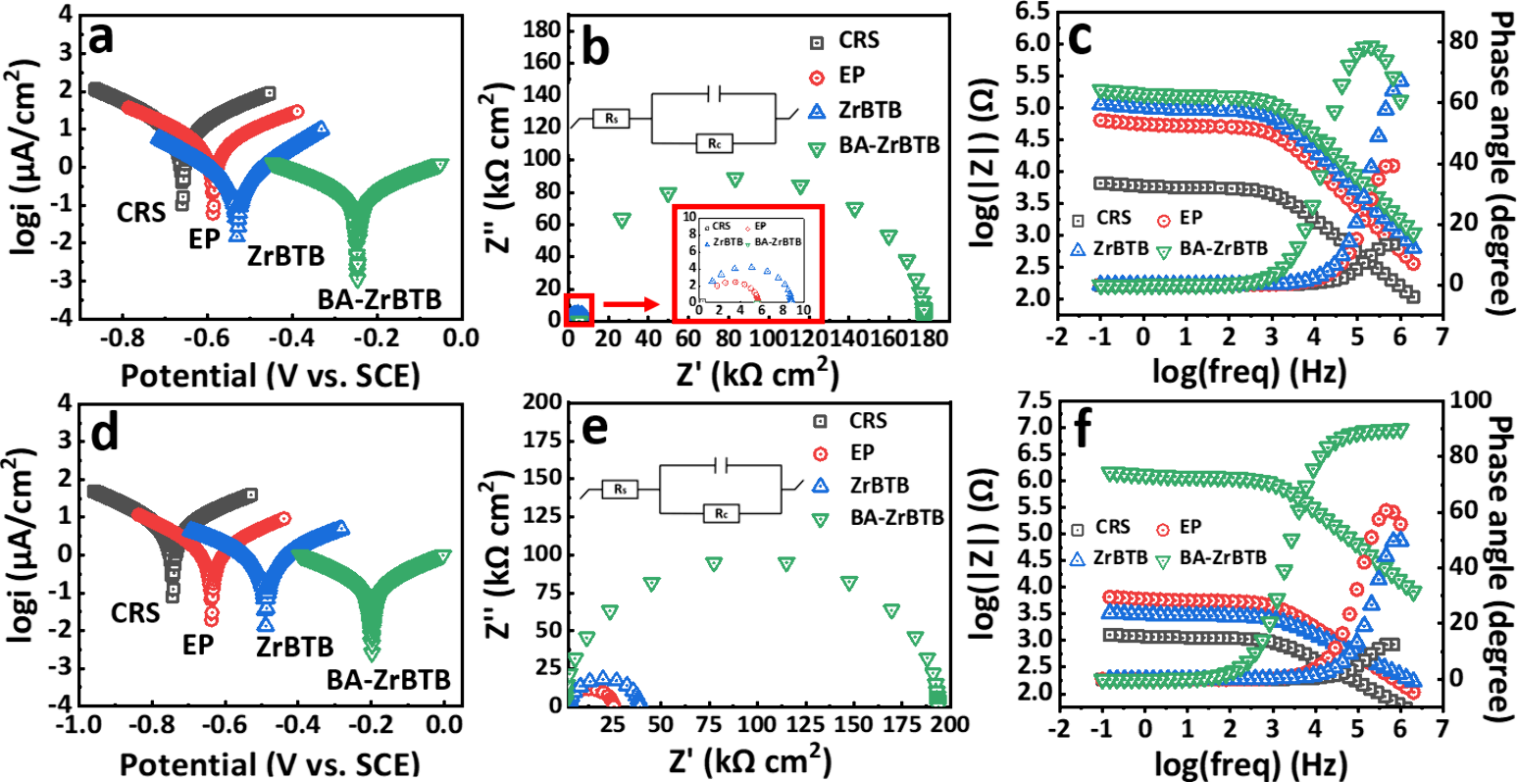
Tafel plots, Nyquist plots, and Bode plots with phase angles for CRS, EP, BA-ZrBTB/EP, and ZrBTB/EP-coated CRS electrodes at 25 ± 0.5 °C measured in 3.5 wt % NaCl aqueous solution for (a–c) 30 min and (d–f) 24 h.

The electrochemical testing encompassed both short-term and long-term immersion of the samples, with durations of 30 min and 24 h, respectively. Figures 6a and 6d present the Tafel plots for CRS, EP, BA-ZrBTB/EP, and ZrBTB/EP coatings, illustrating their anticorrosion characteristics. Furthermore, it is essential to consider the electrochemical performances of the critical materials, ZrO_2_ and H_3_BTB, which are pivotal for synthesizing Zr-MOFs. These performances are depicted in Figure S4, and the tested data are displayed in Tables S1–S4. In the case of CRS, a corrosion current density (*i*_corr_) of 10.23 μA/cm^2^ was recorded after immersing the substrate in a NaCl solution for 30 min (Table 1), indicating inadequate anticorrosion performance for the iron steel substrate. Moreover, significant differences in *i*_corr_ values were observed among EP, BA-ZrBTB/EP, and ZrBTB/EP, with measurements of 3.99, 0.29, and 3.02 μA/cm^2^, respectively. Notably, BA-ZrBTB/EP exhibited the lowest *i*_corr_ value, signifying its outstanding anticorrosion performance. Long-term immersion, involving a 24 h duration of soaking, was also conducted to assess the anticorrosion performance of the materials. This extended immersion study is typically preferred for providing a more stable and precise evaluation of the anticorrosion properties of the materials. After 24 h of immersion, the *i*_corr_ values were measured as follows: 8.92 μA/cm^2^ for CRS, 3.08 μA/cm^2^ for EP, 0.24 μA/cm^2^ for BA-ZrBTB/EP, and 2.26 μA/cm^2^ for ZrBTB/EP. The observed reduction in *i*_corr_ values for all samples during the extended immersion can be attributed to the gradual formation of a protective passive layer on the surface of the material over time. This phenomenon significantly contributes to the enhancement of the material’s anticorrosion properties. Additionally, the corrosion potential (*E*_corr_), representing the corrosion potential of all samples, was measured under identical conditions, resulting in values of −0.661 V for CRS, −0.586 V for EP, −0.246 V for BA-ZrBTB/EP, and −0.490 V for ZrBTB/EP after a 30 min immersion period. The *E*_corr_ values indicated that BA-ZrBTB/EP had the least negative value, signifying the presence of the least spontaneous corrosion reaction. This aligns with the lowest *i*_corr_ values observed for BA-ZrBTB/EP in both short- and long-term immersion studies. Moreover, in the long-term immersion study, after immersing all samples for 24 h, the *E*_corr_ values were measured as follows: −0.783 V for CRS, −0.640 V for EP, −0.198 V for BA-ZrBTB/EP, and −0.469 V for ZrBTB/EP. The negative shift in *E*_corr_ values for CRS and EP coatings implies the formation of corrosion products on the surface, resulting in a shift toward a more negative corrosion potential. Conversely, a positive shift in *E*_corr_ values indicated that the coatings such as BA-ZrBTB/EP and ZrBTB/EP were less affected by the corrosion reaction, signifying superior anticorrosion performance. Furthermore, it was noteworthy that even after 24 h of immersion, BA-ZrBTB/EP continues to exhibit a pronounced anticorrosive trend and the least negative *E*_corr_ value among all samples, underscoring its remarkable corrosion resistance. These findings align with the *i*_corr_ measurements and provide further evidence of the potential of BA-ZrBTB as an effective anticorrosion coating material.

**Table 1:** Corrosion Parameters of the Various Samples Immersed for 30 min in 3.5 wt % NaCl Solution

samples	*E*_corr_ (V)	*i*_corr_ (μA/cm^2^)	*r*_corr_ (mpy)	PE (%)
CRS	–0.661	10.23	175.86	
EP	–0.586	3.99	30.14	61.0
ZrBTB/EP	–0.490	3.02	19.57	70.5
BA-ZrBTB/EP	–0.246	0.29	3.3 × 10^–3^	97.2

Tables 1 and 2 display the polarization parameters, which encompass *E*_corr_ (V), *i*_corr_ (μA/cm^2^), *r*_corr_ (mpy), and PE (%). The calculation of *r*_corr_ was carried out in accordance with eq 1:^59−62^
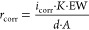
1In eq 1, *K* represents the constant defining the units of the corrosion rate, EW stands for equivalent weight (g/equiv), *d* is the density (g/cm^3^), and *A* represents the sample area (cm^2^). Following the immersion of all samples in a NaCl solution for 30 min, the *r*_corr_ for CRS was calculated as 175.86 mpy, whereas the *r*_corr_ for BA-ZrBTB/EP was 3.3 × 10^–3^ mpy. The substantial discrepancy in corrosion rates between these two samples can be attributed to the higher *i*_corr_ and lower protective capacity of the CRS substrate. This emphasized the effectiveness of BA-ZrBTB as a corrosion-resistant material. Upon extending the immersion time to 24 h, the *r*_corr_ values for CRS, EP, BA-ZrBTB/EP, and ZrBTB/EP coatings notably decreased to 91.74, 4.71, 2.28 × 10^–3^, and 10.48 mpy, respectively. Importantly, the BA-ZrBTB/EP coating exhibited the lowest *r*_corr_ value among all the coated samples. The spatially dispersed 2D sheets of Zr-MOFs provided a substantial external surface area for effective adhesion to the substrate and can also serve as a physical barrier to provide further protection against corrosion due to its higher aspect ratio compared to 3D structures. Table 1 illustrates the calculation of the protection efficiency (PE%) using eq 2:^63−65^
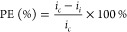
2In eq 2, *i*_c_ represents the corrosion current density of the CRS substrate, while *i*_*i*_ represents the corrosion current density of the composite coatings on the CRS surface. After immersing all samples for 30 min (short period), the calculated PE values are as follows: EP at 61.0%, BA-ZrBTB/EP at 97.2%, and ZrBTB/EP at 70.5%. These values reflect the anticorrosion performance during a brief exposure to the corrosive environment. Upon extending the immersion to 24 h (long period), the PE of the EP coating measures 65.5%, indicating moderate anticorrosion performance over prolonged exposure to the corrosive environment. However, BA-ZrBTB/EP still maintains a high PE value of 97.3%. The higher *E*_corr_ and lower *i*_corr_ and *r*_corr_ values of BA-ZrBTB/EP after 24 h of immersion indicate the stability of its water-stable anticorrosion structure, showcasing the best corrosion resistance among all samples. To validate the results of the Tafel plots, Nyquist and Bode plots were employed. An equivalent circuit model was employed to fit the experimental data, as shown in the inset of Figure 6b,e. This analysis enabled the determination of various parameters, including solution resistance (*R*_s_),^67,68^ film resistance (*R*_c_), and total resistance (*R*_t_), listed in Tables 3 and 4. In Figure 6b, *R*_t_ values for CRS, EP, BA-ZrBTB/EP, and ZrBTB/EP were measured as 0.44, 5.10, 178.05, and 8.95 kΩ·cm^2^, respectively. Besides that, the impedance of ZrO_2_/EP and H_3_BTB/EP coatings was also evaluated and is presented in Figure S4b,e. According to the results, BA-ZrBTB/EP exhibited the highest resistance during short-term immersion tests. It is important to validate the trend of electrochemical impedance over a longer period, as long-term analysis provides higher reliability. There is the possibility that the protective properties of BA-ZrBTB/EP degrade or become compromised under prolonged exposure to corrosive environments. As shown in Figure 6e, the long-term immersion test revealed that the semicircles of all samples remained intact, demonstrating long-term stability and resistance to corrosion. The *R*_t_ value for BA-ZrBTB/EP was measured at 180.80 kΩ·cm^2^, approximately 600 times higher than that of the CRS substrate and 4.7 times higher than that of the ZrBTB/EP coating. These results emphasize that BA-ZrBTB/EP offers exceptional corrosion protection even during extended exposure to corrosive environments.

**Table 2:** Corrosion Parameters of the Various Samples Immersed for 24 h in 3.5 wt % NaCl Solution

samples	*E*_corr_ (V)	*i*_corr_ (μA/cm^2^)	*r*_corr_ (mpy)	PE (%)
CRS	–0.738	8.92	91.74	
EP	–0.640	3.08	4.71	65.5
ZrBTB/EP	–0.469	2.26	10.48	88.6
BA-ZrBTB/EP	–0.198	0.24	2.28 × 10^–3^	97.3

**Table 3:** Simulation Parameters for the Equivalent Circuit Diagrams of Various Samples Immersed for 30 min

samples	*R*_s_ (kΩ·cm^2^)	*R*_c_ (kΩ·cm^2^)	*R*_t_ (kΩ·cm^2^)
CRS	0.03	0.41	0.44
EP	0.17	5.80	5.97
ZrBTB/EP	0.41	8.54	8.95
BA-ZrBTB/EP	2.41	178.39	180.80

**Table 4:** Simulation Parameters for the Equivalent Circuit Diagrams of Various Samples Immersed for 24 h

samples	*R*_s_ (kΩ·cm^2^)	*R*_c_ (kΩ·cm^2^)	*R*_t_ (kΩ·cm^2^)
CRS	0.03	0.30	0.33
EP	0.37	25.85	26.22
ZrBTB/EP	0.48	40.98	41.26
BA-ZrBTB/EP	3.73	193.59	197.32

The assessment of coating protective properties is commonly conducted by analyzing its Bode plots and phase angle, as depicted in Figure 6c,f. In the short-term test, BA-ZrBTB/EP composite coatings exhibited superior corrosion resistance compared with the other samples, as evidenced by their higher impedance value at 10 mHz. Furthermore, BA-ZrBTB/EP also exhibited the highest phase angle and the slowest decline rate with varying log(frequency) (Hz). These results underscored the efficacy of BA-ZrBTB as a protective coating material for corrosion prevention. Similarly, after prolonged immersion in a 3.5 wt % NaCl solution, BA-ZrBTB/EP continued to demonstrate the highest corrosion resistance in the Bode plots and the highest phase angle, as illustrated in Figure 6f. The elevated impedance values observed for BA-ZrBTB/EP composite coatings can be attributed to the exceptional barrier properties conferred by the incorporation of 2D Zr-MOFs with benzoate moieties. Within the MOF structure, the presence of benzoate linkages facilitates the formation of a protective film, effectively impeding direct contact between the metal substrate and corrosive environments and thereby mitigating corrosion.

On the other hand, Bode plots and phase angles of ZrO_2_/EP and H_3_BTB/EP are also displayed in Figures S4c and S4f, respectively, illustrating the higher protection of coating due to the corresponding of higher impedance value which provides from the dense and protective oxide layer upon exposure to corrosive environments. This oxide layer acts as a barrier, preventing direct contact between the underlying material and the corrosive medium, thus slowing down the corrosion process and showing great anticorrosion electrochemical performances such as high *E*_corr_ and low *i*_corr_ and *r*_corr_.^50,51^ In conclusion, the results of the electrochemical tests substantiate the potential of BA-ZrBTB/EP composite coatings as a promising material for anticorrosion applications across various industries.

**Figure 7:**
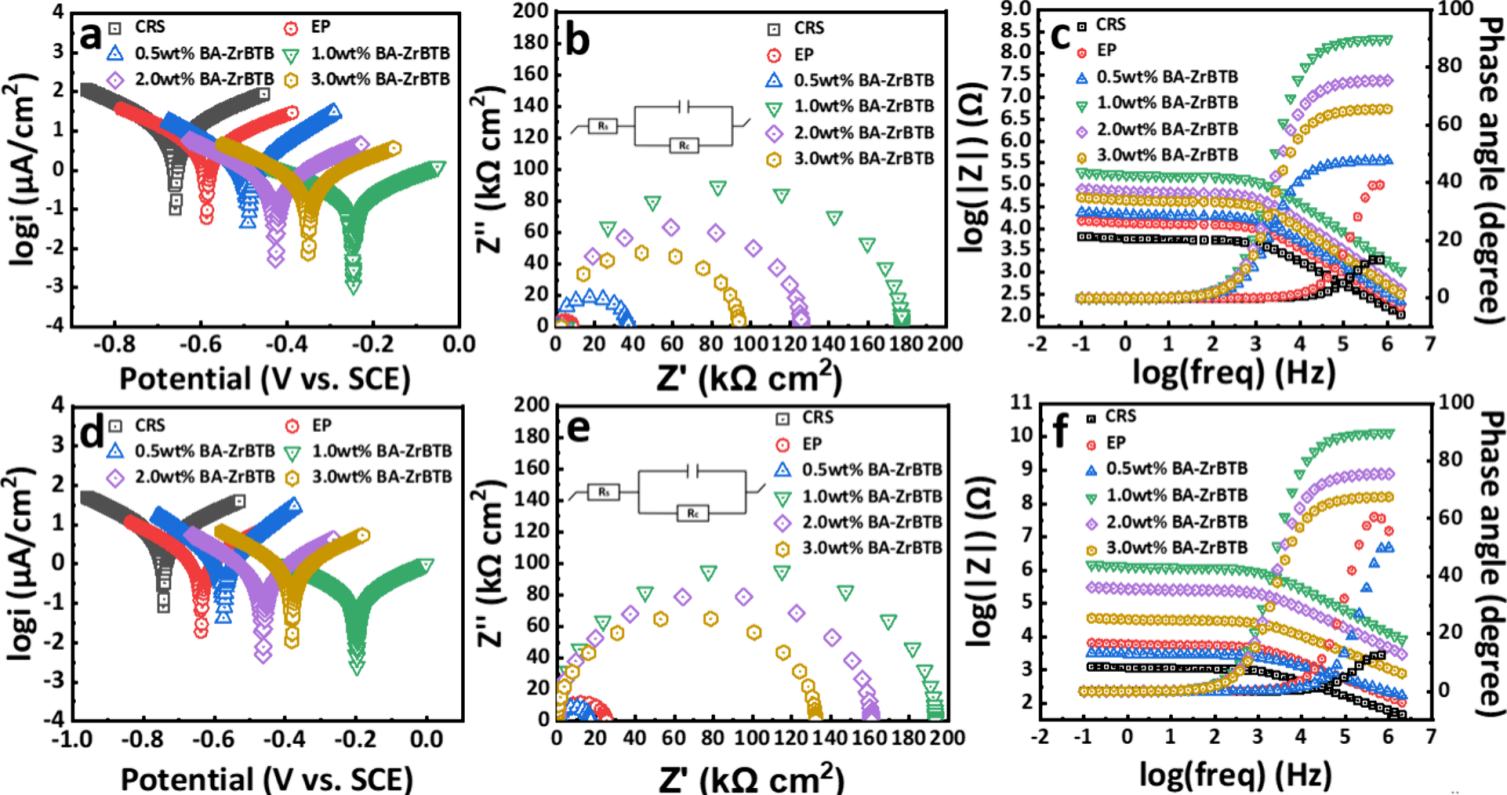
Tafel plots, Nyquist plots, and Bode plots with phase angles for various concentrations of BA-ZrBTB composite coatings at a temperature of 25 ± 0.5 °C, measured in a 3.5 wt % NaCl aqueous solution for (a–c) 30 min and (d–f) 24 h.

To optimize the anticorrosion efficiency, various BA-ZrBTB solid contents, specifically 0.5, 1.0, 2.0, and 3.0 wt %, were tested and compared before evaluating the performance of CRS, EP, BA-ZrBTB/EP, and ZrBTB/EP composite coatings. As shown in Figure 7, the coating containing 1.0 wt % BA-ZrBTB exhibited the most favorable results, including the lowest *i*_corr_ (0.24 μA/cm^2^), the lowest *r*_corr_ (2.28 × 10^–3^ mpy), the highest *E*_corr_ (−0.198 V), the highest corrosion impedance (197.32 kΩ·cm^2^), and the most significant level of coating protection, as analyzed by Bode plots and phase angles after 24 h of immersion in NaCl solution. It was observed that a lower content of BA-ZrBTB additive, such as 0.5 wt %, resulted in an uneven coating due to the insufficient content, which could not fully harness the intended benefits of BA-ZrBTB as an additive. Conversely, the reason higher BA-ZrBTB solid content does not lead to improved anticorrosion performance is due to several factors. First, an excess of particles can result in a “filling effect” within the coating, reducing its density and potentially increasing the chances of oxygen or moisture penetration, thereby weakening its corrosion protection. Moreover, issues related to particle dispersibility may arise with excessive particle addition, leading to aggregation or uneven distribution. This can result in the formation of pores and defects within the coating, increasing the risk of corrosion. Furthermore, an excessively thick coating, caused by high particle loading, can limit direct contact between the substrate and the corrosive medium, diminishing the material’s overall corrosion resistance. Lastly, the use of additional binding agents to accommodate excessive particles may affect the overall stability of the coating. Therefore, achieving the optimal balance of particle content in the design of corrosion-resistant coatings is crucial to ensuring their effectiveness. Excessive particle loading may have adverse effects, compromising corrosion resistance rather than improving corrosion resistance. On the other hand, the long-term open circuit potential (OCP) tests of CRS and different coating samples have been conducted and are presented as Figure S5. These tests aim to observe the stability and long-term electrochemical performance of CRS and coating samples. Initially, the CRS substrate exhibits the lowest potential at −0.698 V, indicating the maximum spontaneous corrosion activity. In contrast, the BA-ZrBTB/EP sample shows a higher initial potential of −0.341 V, demonstrating superior performance compared to CRS. Over 24 h, CRS experiences a significant potential drop from −0.698 to −0.882 V, indicative of substantial corrosion. Conversely, the BA-ZrBTB/EP potential remains stable around −0.325 V after 24 h, underscoring its effective barrier properties. These results after 8 days highlight the anticorrosion advantages and long-term stability of BA-ZrBTB/EP and ZrBTB/EP coatings.

**Figure 8:**
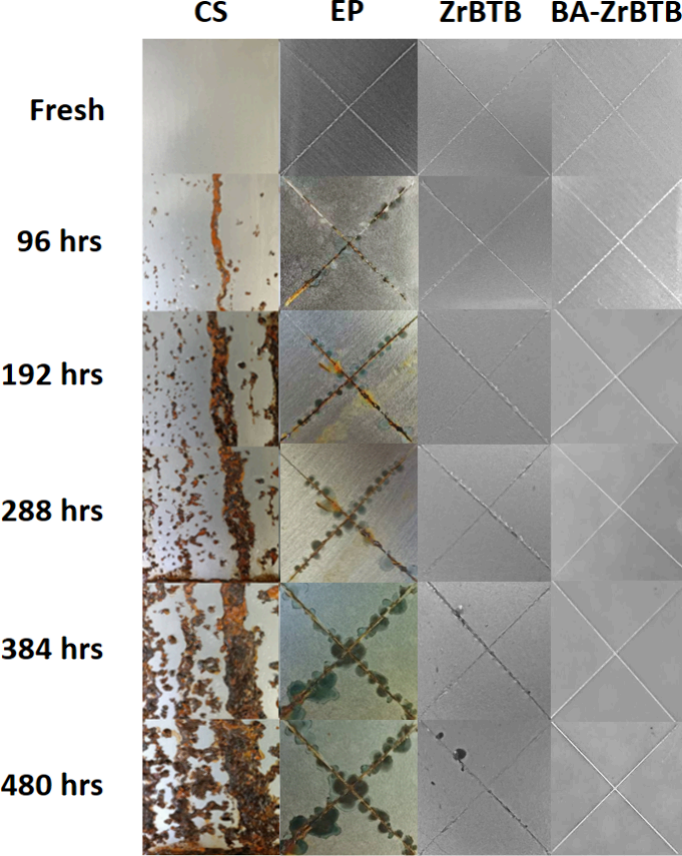
Photo images of CS, EP, ZrBTB/EP, and BA-ZrBTB/EP after salt spray corrosion tests for 480 h.

The nanocomposite coatings underwent salt spray tests, in which fresh and coated plates were exposed to a saline fog chamber. The test results, shown in Figure 8, were observed over a period of up to 480 h (20 days) of exposure. The samples included carbon steel (CS), EP, ZrBTB/EP, and BA-ZrBTB/EP. A 5 × 5 cm^2^ scribed region was applied to each sample, except for CS, which was left blank, as it could fully rust within a few hours. After 96 h of exposure, rust was evident on the CS and EP samples due to their weak protective ability. As the spraying continued for 288 h, dark traces of corrosion appeared near the scribed region, from the top left to the bottom right of the ZrBTB/EP coating. However, the BA-ZrBTB/EP coating remained intact, indicating its superior corrosion resistance. Nevertheless, after 480 h of exposure to salt spray, the scribed region of BA-ZrBTB/EP began to show signs of penetration and rust started to appear. This suggests that while BA-ZrBTB/EP may not be completely immune to corrosion under extreme environmental conditions, it still offers excellent corrosion resistance over an extended period.

**Figure 9:**
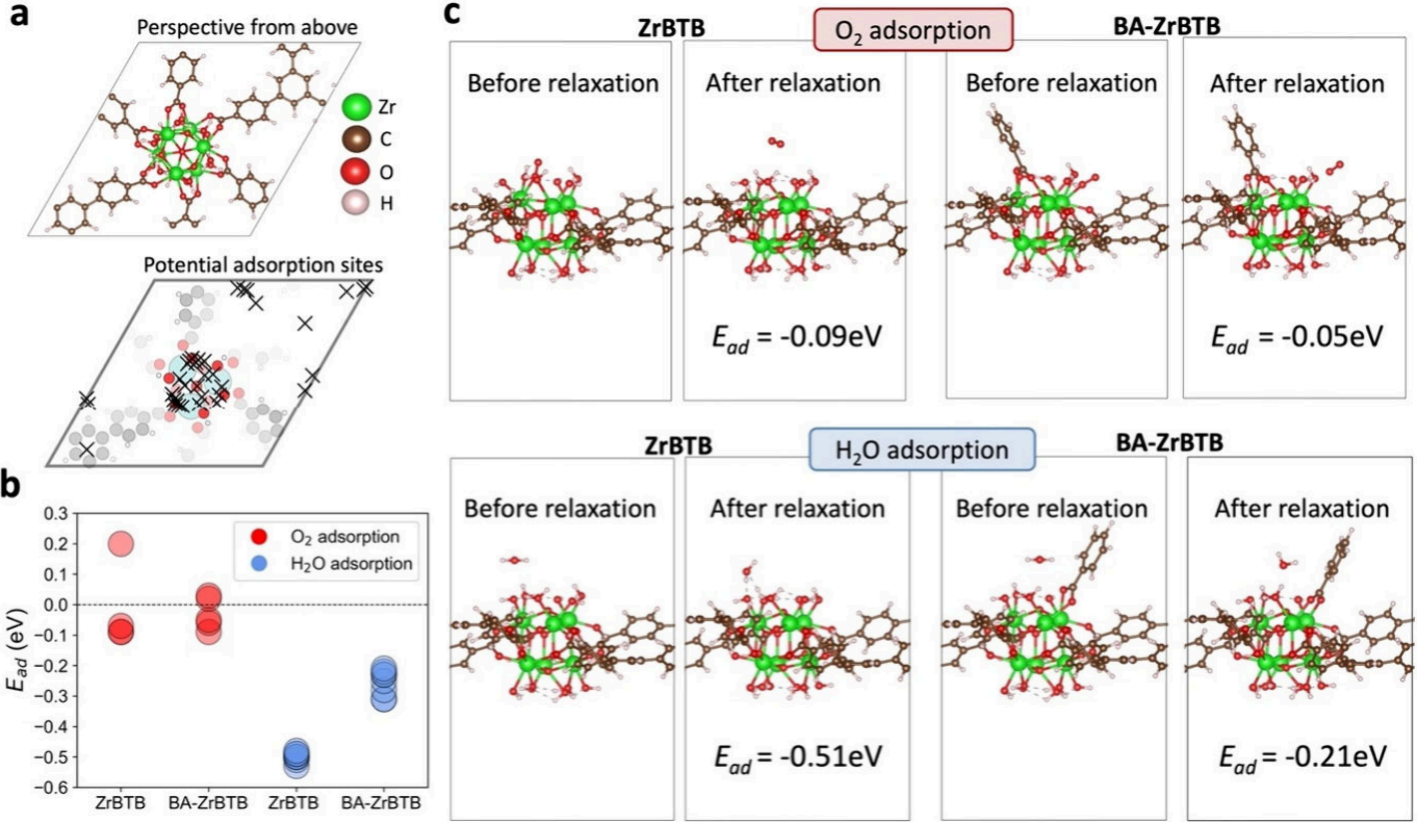
(a) Overhead view of the ZrBTB surface highlighting potential adsorption sites (marked with cross signs) identified using the Delaunay triangulation method,^41^ as integrated in the adsorption module of Pymatgen.^42^ (b) DFT-calculated adsorption energies (*E*_ad_) for ZrBTB and BA-ZrBTB surfaces with optimally oriented O_2_ and H_2_O molecules, respectively. These orientations were identified through screening by the fine-tuned machine-learning based interatomic potential, CHGNet.^43^ Each circle denotes a distinct data point, with darker shades indicating higher data point overlap. (c) Comparative visualizations of O_2_ and H_2_O molecules pre- and post-geometry optimization (relaxation) on the ZrBTB and BA-ZrBTB surfaces.

To unravel the underlying reasons and mechanisms behind the superior anticorrosive properties of BA-ZrBTB compared to ZrBTB, we conducted theoretical first-principles calculations for a deeper insight. Given that O_2_ and H_2_O are the primary reactants in corrosion processes, we proposed that the adsorption behaviors of these molecules on ZrBTB and BA-ZrBTB surfaces might significantly differ, contributing to their varying anticorrosion capabilities. Accordingly, we modeled the 2D surface structures of ZrBTB and BA-ZrBTB and utilized the Delaunay triangulation algorithm,^42^ available in the Pymatgen package,^43^ to identify potential adsorption sites, as depicted in Figure 9a. Considering the complex and varied potential orientations of the O_2_ and H_2_O molecules on these surfaces, alongside the substantial atomic count in the models (exceeding 150 atoms), the computational demands for exhaustive orientation calculations could be prohibitively high. To circumvent this, we employed CHGNet,^44^ a universal machine-learning interatomic potential, for an initial rapid assessment of the energy associated with each molecular orientation. However, the initial CHGNet predictions could be skewed due to the absence of ZrBTB and BA-ZrBTB surface structures with adsorbed O_2_/H_2_O molecules in its training database (Materials Project).^69^ To address this limitation and enhance the accuracy of our predictions, we carried out density functional theory (DFT) calculations on select surface structures featuring O_2_/H_2_O adsorption. These results were then used to fine-tune the CHGNet model, creating a customized, more precise tool for energy estimation, as illustrated in Table S5. Leveraging this optimized model, we evaluated a total 1,870 different O_2_/H_2_O orientations, selecting those with lower relative energies for subsequent detailed DFT analysis. The CHGNet-calcualted energies of all 1,870 configurations are presented in Figure S6.

Figure 9b illustrates the DFT-calculated adsorption energies (*E*_ad_) of O_2_ and H_2_O on the ZrBTB and BA-ZrBTB surfaces. The adsorption energy for O_2_ molecules on both ZrBTB and BA-ZrBTB surfaces ranges from −0.1 to 0.2 eV, indicating a weak or nonfavorable adsorption affinity. In contrast, H_2_O demonstrates a notably more negative adsorption energy on ZrBTB (around −0.5 eV) compared to BA-ZrBTB (between −0.2 and −0.3 eV). The adsorption energy is calculated by subtracting the sum of the pure adsorbate and surface structure energies from the final energy of the adsorbate bound to the MOF, with values near zero or positive, suggesting unfavorable O_2_ adsorption on both MOFs. These results indicated low O_2_ permeability in both ZrBTB and BA-ZrBTB, indicating their resistance to O_2_ infiltration. The considerable difference in H_2_O adsorption energies between the two MOFs potentially underpins BA-ZrBTB’s enhanced anticorrosive properties. The stronger H_2_O adsorption on ZrBTB, as evidenced by its more negative adsorption energy, contrasts with the weaker interaction observed on BA-ZrBTB. Figure 9c presents the adsorption configurations of O_2_ and H_2_O molecules before and after geometry optimization via DFT. Post-optimization, O_2_ molecules are positioned further from both MOF surfaces, demonstrating their resistance to O_2_. Meanwhile, H_2_O molecules show potential coordination with −OH and −OH_2_ groups on ZrBTB’s surface. However, such stable attachment is not observed on BA-ZrBTB, likely due to steric hindrance and repulsive forces introduced by benzoic acid, which impedes H_2_O molecule adsorption. All of the other adsorption structures are detailed in Figures S7–S10. Through comprehensive theoretical analysis, we propose that BA-ZrBTB’s resistance to both O_2_ and H_2_O contributes to its superior anticorrosive performance.

**Figure 10:**
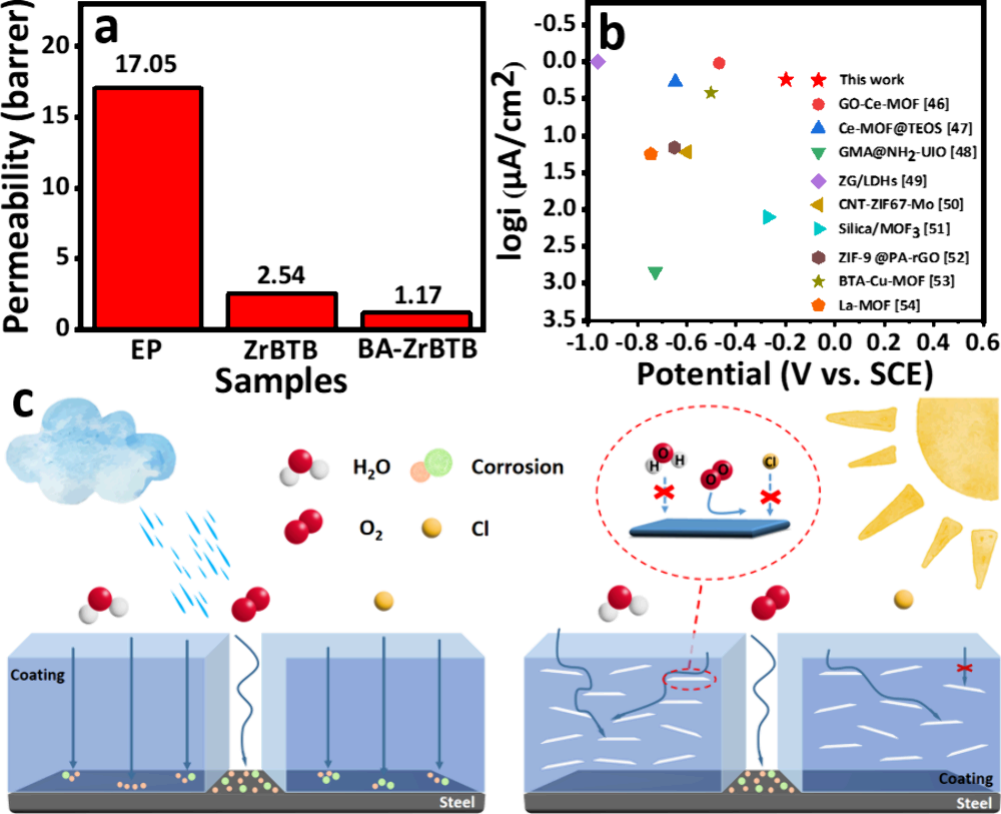
(a) Permeability tests of oxygen in EP, ZrBTB/EP, and BA-ZrBTB/EP coatings. (b) Comparison of anticorrosive coatings of our work and reported references. (c) Anticorrosion mechanism of EP and BA-ZrBTB/EP coatings.

Figure 10a illustrates the results of the gas permeability analysis by evaluating the oxygen gas (O_2_) permeability in these coatings. The analysis revealed that the EP coating had a relatively high O_2_ permeability of 17.05 barrer, which was possibly due to the drying process employed during the coating preparation. When the solvent rapidly evaporated due to the high temperature during the coating preparation, the pores in the epoxy resin were preserved. These pores can form a pathway for O_2_ to permeate through the coating. However, the incorporation of BA-ZrBTB and ZrBTB additives results in a notable reduction in the permeability of the O_2_ to 1.17 and 2.54 barrer, respectively. This decrease may be attributed to the spatially dispersed and tortuous arrangement of 2D MOF sheets with a high aspect ratio. Such a tortuous arrangement significantly hampered the path of gas permeation, thus diminishing the likelihood of corrosion occurrence. Moreover, it was noteworthy that the O_2_ permeability values of BA-ZrBTB and ZrBTB were comparable but markedly lower than that of the EP sample, indicating the presence of Zr-based MOFs with a 2D structure, which impart superior gas barrier properties. Consequently, the formation of a dense and uniform protective coating becomes imperative, serving as a robust barrier against the ingress of gas molecules upon exposure to water and corrosive ions. The experimental results were in consistent with DFT results shown in Figure 9.

High gas permeability in EP can lead to the formation of microchannels or defects within the coating, providing a pathway for corrosive agents to reach the metal surface and initiate corrosion. Moreover, MOF-based coatings with low gas permeability effectively block the diffusion of corrosive agents, thus enhancing the protection of the underlying substrate against corrosion. To verify the anticorrosion performance of the BA-ZrBTB/EP coating, it was also compared with those of other MOF-based anticorrosive coatings reported in the literature, and the results are shown in Figure 10b. The results clearly highlight the exceptional anticorrosion performance of the 2D BA-ZrBTB/EP coating when compared to other coatings.

In conclusion, Figure 10c illustrates the corrosion and anticorrosion mechanisms of the EP and BA-ZrBTB/EP coatings, corresponding to the left and right figures, respectively. The middle of the coating is left blank, where corrosion ions, such as water (H_2_O), O_2_, and Cl ions, can easily attach to the substrate, leading to serious corrosion, similar to that of the EP coating region. In the case of the BA-ZrBTB coating, when corrosion ions permeate the coating and attach to the uniform 2D hydrophobic BA-ZrBTB layer structure, the corrosion ions are forced to detour. This significant detour delays the corrosion reaction occurring on the substrate surface due to the high aspect ratio and the special anticorrosive characteristics of Zr-MOFs.

## 4 Conclusions

2D zirconium-based MOFs with and without benzoate ligands coordinated on their hexazirconium nodes can be successfully synthesized, and EP-based composite coatings with these 2D Zr-MOFs as additives can be fabricated on the surface of CS substrates. The results confirm the uniform distributions of carbon (C), oxygen (O), and zirconium (Zr) elements both within the BA-ZrBTB/EP coatings. Remarkably, the hydrophobic BA-ZrBTB/EP composite coatings exhibit significantly enhanced electrochemical resistance compared to EP, with resistance measured at 197.32 kΩ·cm^2^ after 24 h of immersion in NaCl solution, in contrast to the resistance of 26.22 kΩ·cm^2^ of EP coating. Moreover, the 480 h of salt spray test for BA-ZrBTB/EP revealed the highly protective composite coating ability to effectively block the permeation of corrosive ions, demonstrating excellent corrosion resistant properties. Additionally, both BA-ZrBTB/EP and ZrBTB/EP exhibit much lower oxygen permeability compared with EP coatings. This enhanced resistance was attributed to the high energy barrier and the high aspect ratio inherent in the 2D structure of the additive. These characteristics position BA-ZrBTB as a promising candidate for durable and long-lasting materials in anticorrosion applications. Indeed, it is worth highlighting that this study represents the first application of 2D Zr-MOFs as additive materials in anticorrosion applications.

## Supporting Information Available

Additional data of Figure S1: XRD patterns of (a) H3BTB and (b) ZrO_2_ powders. Figure S2: (a) SEM images at a low magnification with an inset showing the EDS elemental mapping for Zr and (b) high-magnification SEM image of ZrBTB; (c) low-magnification TEM image; (d) EDS elemental mapping for Zr collected from the rectangular region shown in (c) and (e) HR-TEM image of the ZrBTB nanosheet; (f) HAADF-STEM image of the single ZrBTB sheet showing lattice fringes of the 2D MOF; crystalline structure of ZrBTB with one layer is shown in the inset of (f). Figure S3: SEM images of (a) BA-ZrBTB and (b) ZrBTB; TEM images of (c) BA-ZrBTB and (d) ZrBTB. Figure S4: Tafel plots, Nyquist plots, and Bode plots with phase angles for CRS, EP, ZrO_2_/EP, H_3_BTB/EP, and BA-ZrBTB/EP-coated CRS electrodes at 25 ± 0.5 °C measured in 3.5 wt % NaCl aqueous solution for (a–c) 30 min and (d–f) 24 h. Figure S5: long-term open circuit potential (OCP) tests of CRS and different coating samples. Figure S6: calculated total energy via the fine-tuned CHGNet machine-learning model; the width of the violin plots indicates the relative number of data points. Figure S7: structures optimized via DFT of various initial O_2_ orientations on ZrBHT surfaces, alongside their corresponding adsorption energies (*E*_ad_); the structures labeled with *E*_ad_ values represent the configurations after geometry optimization, while the structure to the left illustrates the orientation prior to optimization. Figure S8: structures optimized via DFT of various initial O_2_ orientations on BA-ZrBHT surfaces, alongside their corresponding adsorption energies (*E*_ad_); the structures labeled with *E*_ad_ values represent the configurations after geometry optimization, while the structure to the left illustrates the orientation prior to optimization. Figure S9: structures optimized via DFT of various initial H_2_O orientations on ZrBHT surfaces, alongside their corresponding adsorption energies (*E*_ad_); the structures labeled with *E*_ad_ values represent the configurations after geometry optimization, while the structure to the left illustrates the orientation prior to optimization. Figure S10: structures optimized via DFT of various initial H_2_O orientations on BA-ZrBHT surfaces, alongside their corresponding adsorption energies (*E*_ad_); the structures labeled with *E*_ad_ values represent the configurations after geometry optimization, while the structure to the left illustrates the orientation prior to optimization. Table S1: corrosion parameters of the various samples as CRS, EP, ZrO_2_/EP, H_3_BTB/EP, and BA-ZrBTB/EP immersed for 30 min in 3.5 wt % NaCl solution. Table S2: corrosion parameters of the various samples as CRS, EP, ZrO_2_/EP, H_3_BTB/EP, and BA-ZrBTB/EP immersed for 24 h in 3.5 wt % NaCl solution. Table S3: simulation parameters for the equivalent circuit diagrams of various samples as CRS, EP, ZrO_2_/EP, H_3_BTB/EP, and BA-ZrBTB/EP immersing for 30 min. Table S4: simulation parameters for the equivalent circuit diagrams of various samples as CRS, EP, ZrO_2_/EP, H_3_BTB/EP, and BA-ZrBTB/EP immersing for 24 h. Table S5: comparison of total energies calculated using DFT, the CHGNet model (without fine-tuning), and the CHGNet model (fine-tuned with preliminary DFT data) (PDF)
