# Supplementary material for: Two-Dimensional Metal–Organic Frameworks/Epoxy Composite Coatings with Superior O2/H2O Resistance for Anticorrosion Applications
Source: ACS Appl Mater Interfaces. 2024 Jul 12;16(31):41421–34. doi: 10.1021/acsami.4c04843 (PMC11310901; doi:10.1021/acsami.4c04843)
Supplement: Supplementary file 1 — am4c04843_si_001.pdf [file am4c04843_si_001.pdf]

Supporting Information

Two-dimensional Metal–Organic Frameworks/Epoxy Composite Coatings with Superior O_2/H_2O Resistance for Anticorrosion Applications

Hao-Hsuan Hsia,[1,2] You-Liang Chen,[3] Yu-Ting Tai,[3,4] Hong-Kang Tian,[3,4,5] Chung-Wei Kung[3,*] and Wei-Ren Liu[1,*]

[1] Department of Chemical Engineering, R&D Center for Membrane Technology, Research Center for Circular Economy, Chung Yuan Christian University Taoyuan 32023, Taiwan

[2] Department of Graduate Institute of Applied Science and Technology, National Taiwan University of Science and Technology, Taipei City, 106335, Taiwan

[3] Department of Chemical Engineering, National Cheng Kung University, Tainan City, 70101, Taiwan

[4] Program on Smart and Sustainable Manufacturing, Academy of Innovative Semiconductor and Sustainable Manufacturing, National Cheng Kung University, Tainan 70101, Taiwan

[5] Hierarchical Green-Energy Materials (Hi-GEM) Research Center, National Cheng Kung University, Tainan 70101, Taiwan

*Corresponding authors. Tel: +886 3 265 4140;

E-mails: WRLiu1203@gmail.com (W. R. Liu) and cwkung@mail.ncku.edu.tw (C. W. Kung)

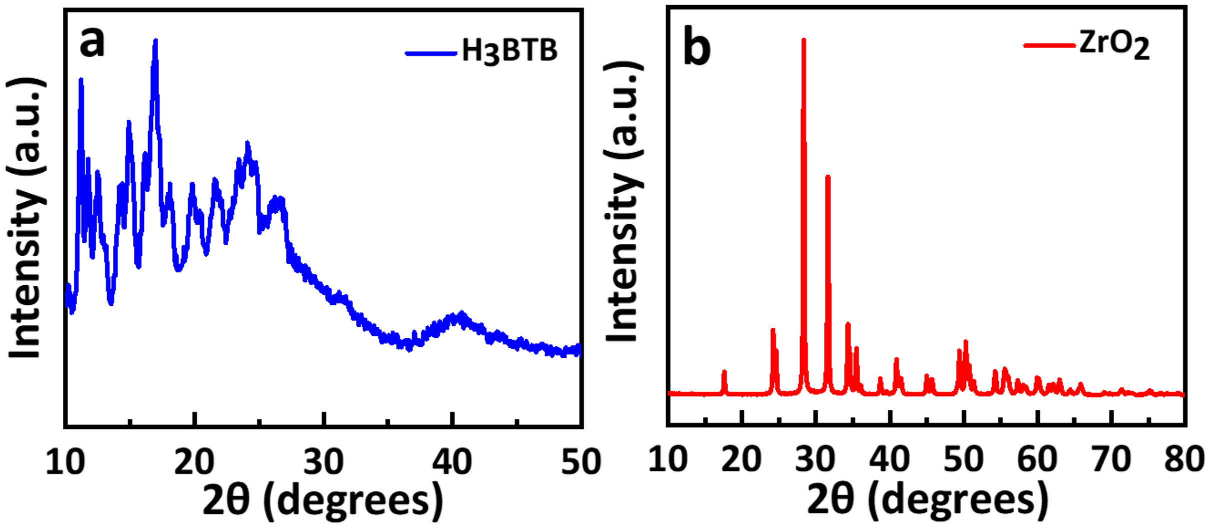

Figure S1. XRD patterns of (a) H_3BTB and (b) ZrO_2 powders.

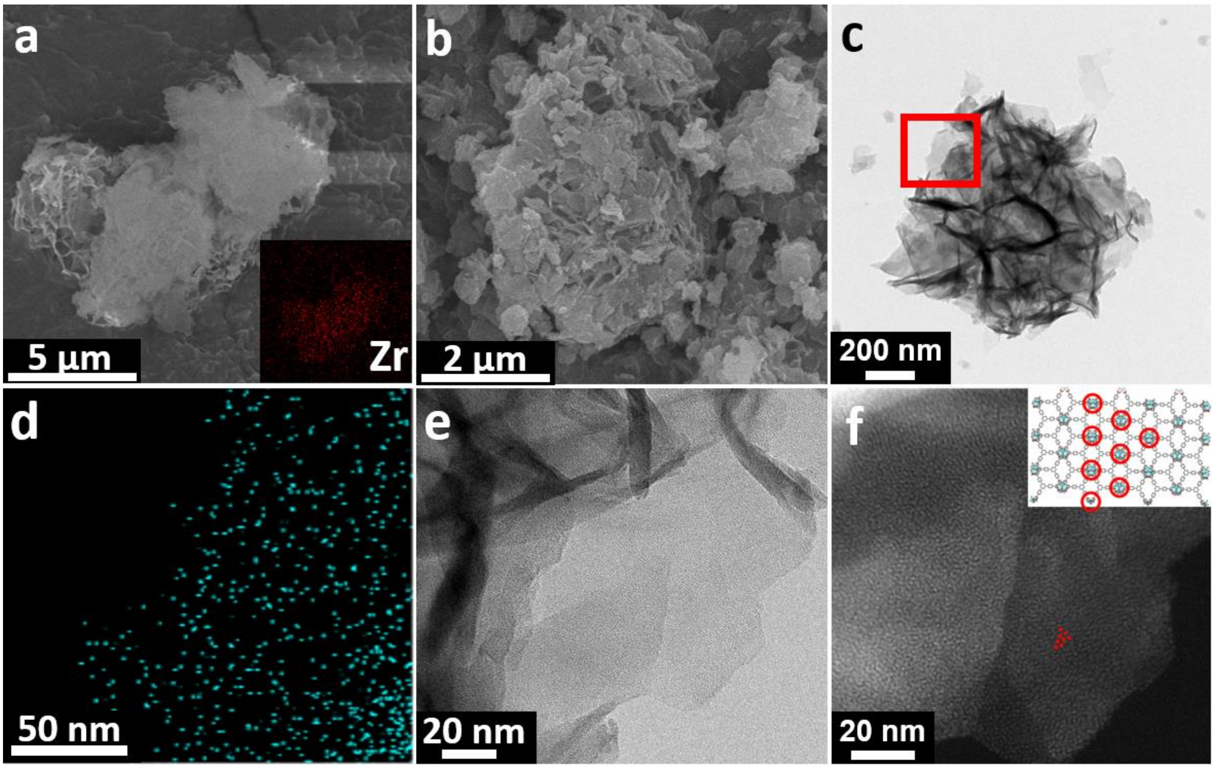

Figure S2. (a) SEM images at a low magnification with an inset showing the EDS elemental mapping for Zr and (b) high-magnification SEM image of ZrBTB. (c) Low-magnification TEM image, (d) EDS elemental mapping for Zr collected from the rectangular region shown in (c) and (e) HR-TEM image of the ZrBTB nanosheet. (f) HAADF-STEM image of the single ZrBTB sheet showing lattice fringes of the 2D MOF. Crystalline structure of ZrBTB with one layer is shown in the inset of (f).

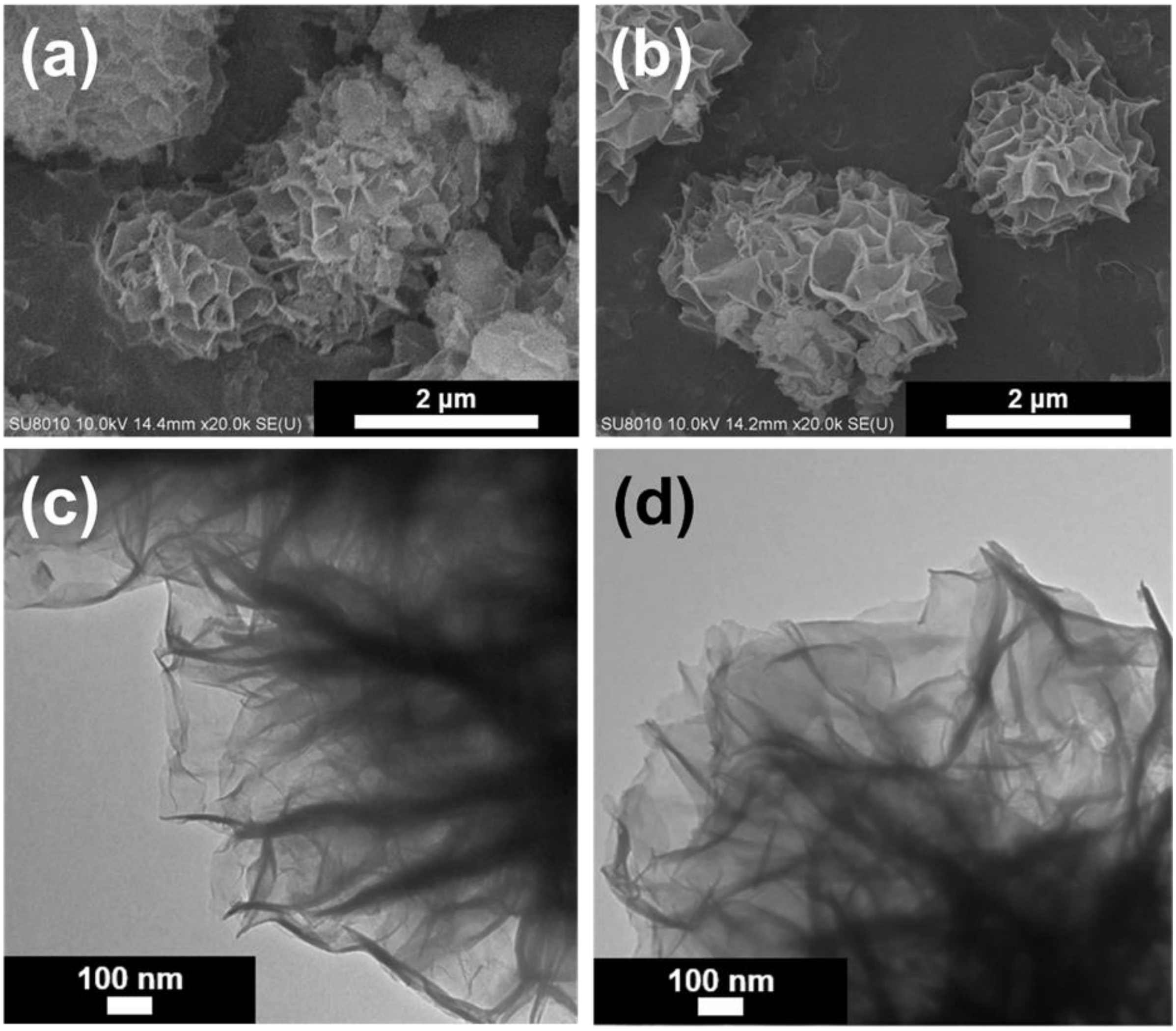

Figure S3. SEM images of (a) BA-ZrBTB and (b) ZrBTB. TEM images of (c) BA-ZrBTB and (d) ZrBTB.

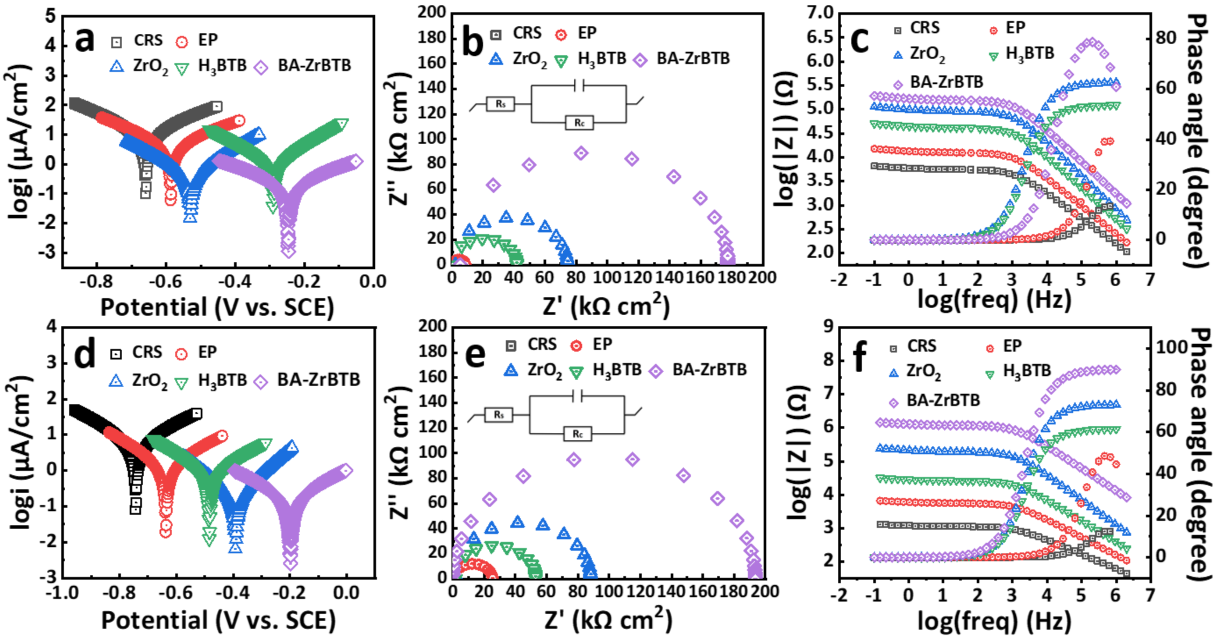

Figure S4. Tafel plots, Nyquist plots and Bode plots with Phase angles for CRS, EP, ZrO_2/EP, H_3BTB/EP and BA-ZrBTB/EP-coated CRS electrodes at 25 ± 0.5°C measured in 3.5 wt% NaCl aqueous solution for (a-c) 30 minutes and (d-f) 24 hours.

Table S1. Corrosion parameters of the various samples as CRS, EP, ZrO_2/EP, H_3BTB/EP and BA-ZrBTB/EP immersed for 30 minutes in 3.5 wt% NaCl solution.

Samples	E_{corr}(V)	i_{corr}(μA/cm^2)	r_{corr}(mpy)	PE(%)
**CRS**	-0.661	10.23	175.86	--
**EP**	-0.586	3.99	30.14	61.0%
**ZrO_2/EP**	-0.538	1.41	9.78	86.2%
**H_3BTB/EP**	-0.292	3.57	20.34	65.1%
**BA-ZrBTB/EP**	-0.246	0.29	$3.3*10^{-3}$	97.2%

Table S2. Corrosion parameters of the various samples as CRS, EP, ZrO_2/EP, H_3BTB/EP and BA-ZrBTB/EP immersed for 24 hours in 3.5 wt% NaCl solution.

Samples	E_{corr}(V)	i_{corr}(μA/cm^2)	r_{corr}(mpy)	PE(%)
**CRS**	-0.738	8.92	91.74	--
**EP**	-0.640	3.08	4.71	65.5%
**ZrO_2/EP**	-0.396	0.97	0.82	89.1%
**H_3BTB/EP**	-0.478	2.63	3.55	70.5%
**BA-ZrBTB/EP**	-0.198	0.24	$2.28*10^{-3}$	97.3%

Table S3. Simulation parameters for the equivalent circuit diagrams of various samples as CRS, EP, ZrO_2/EP, H_3BTB/EP and BA-ZrBTB/EP immersing for 30 minutes.

Samples	R_s (kΩ·cm^2)	R_c (kΩ·cm^2)	R_t (kΩ·cm^2)
**CRS**	0.03	0.41	0.44
**EP**	0.17	5.80	5.97
**ZrO_2/EP**	1.35	76.72	78.07
**H_3BTB/EP**	0.85	41.47	42.32
**BA-ZrBTB/EP**	2.41	178.39	180.80

Table S4. Simulation parameters for the equivalent circuit diagrams of various samples as CRS, EP, ZrO_2/EP, H_3BTB/EP and BA-ZrBTB/EP immersing for 24 hours.

**Samples**	**R_s (kΩ·cm^2)**	**R_c (kΩ·cm^2)**	**R_t (kΩ·cm^2)**
**CRS**	0.03	0.30	0.33
**EP**	0.37	25.85	26.22
**ZrO_2/EP**	1.88	89.62	91.50
**H_3BTB/EP**	0.89	54.22	55.11
**BA-ZrBTB/EP**	3.73	193.59	197.32

Table S5. Comparison of total energies calculated using DFT, the CHGNet model (without fine-tuning), and the CHGNet model (fine-tuned with preliminary DFT data).

	O_2 on ZrBTB	O_2 on BA-ZrBTB	H_2O on ZrBTB	H_2O on BA-ZrBTB
Total energy (eV) (DFT)	**-999.8047**	**-1069.8551**	**-1005.6860**	**-1075.5969**
Total energy (eV) (CHGNet, no fine-tuning)	-1018.0784	-1087.2738	-1023.5657	-1092.7661
Total energy (eV) (CHGNet, fine-tuned with our DFT data)	**-999.9657**	**-1069.2693**	**-1005.3559**	**-1075.3283**

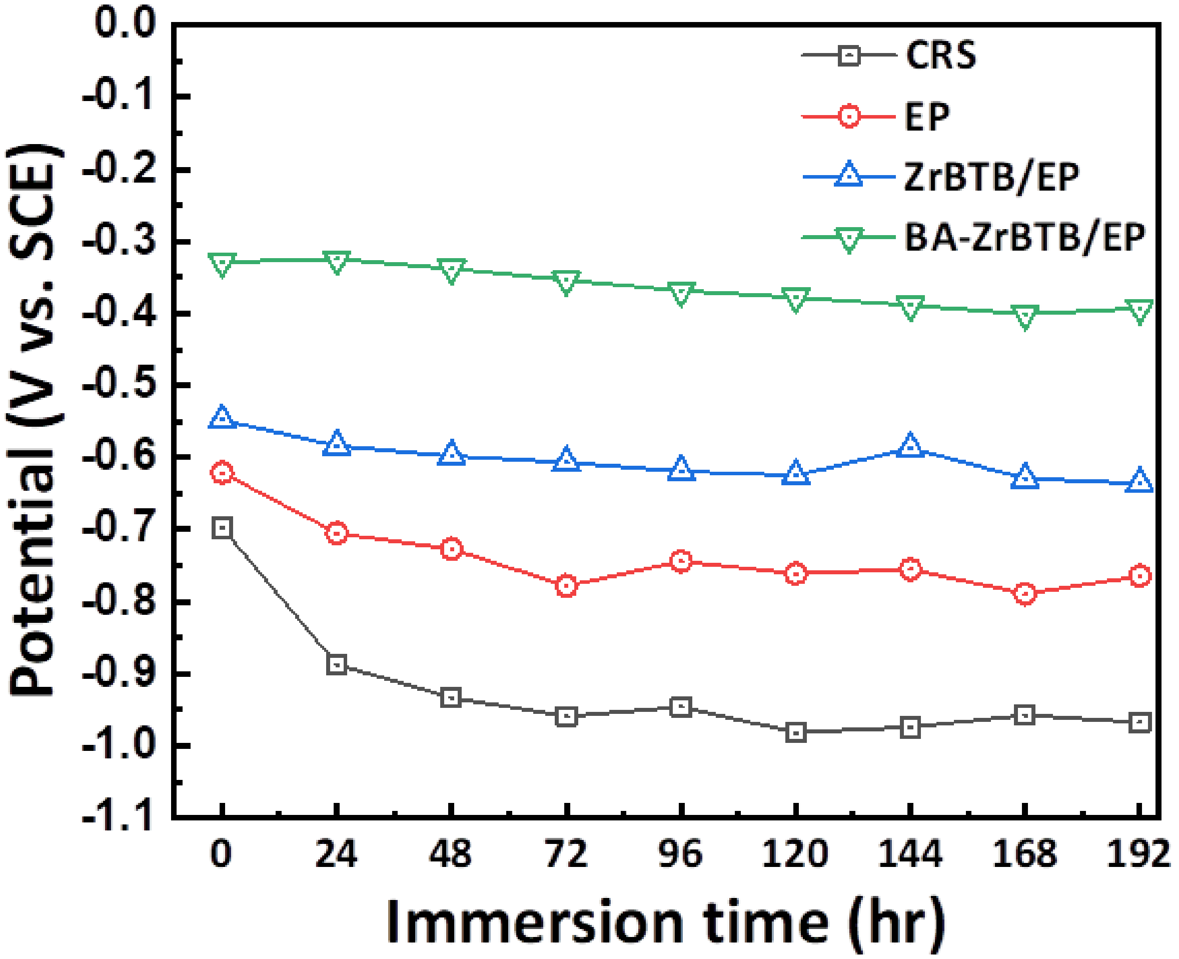

Figure S5. Long-term open circuit potential (OCP) tests of CRS and different coating samples.

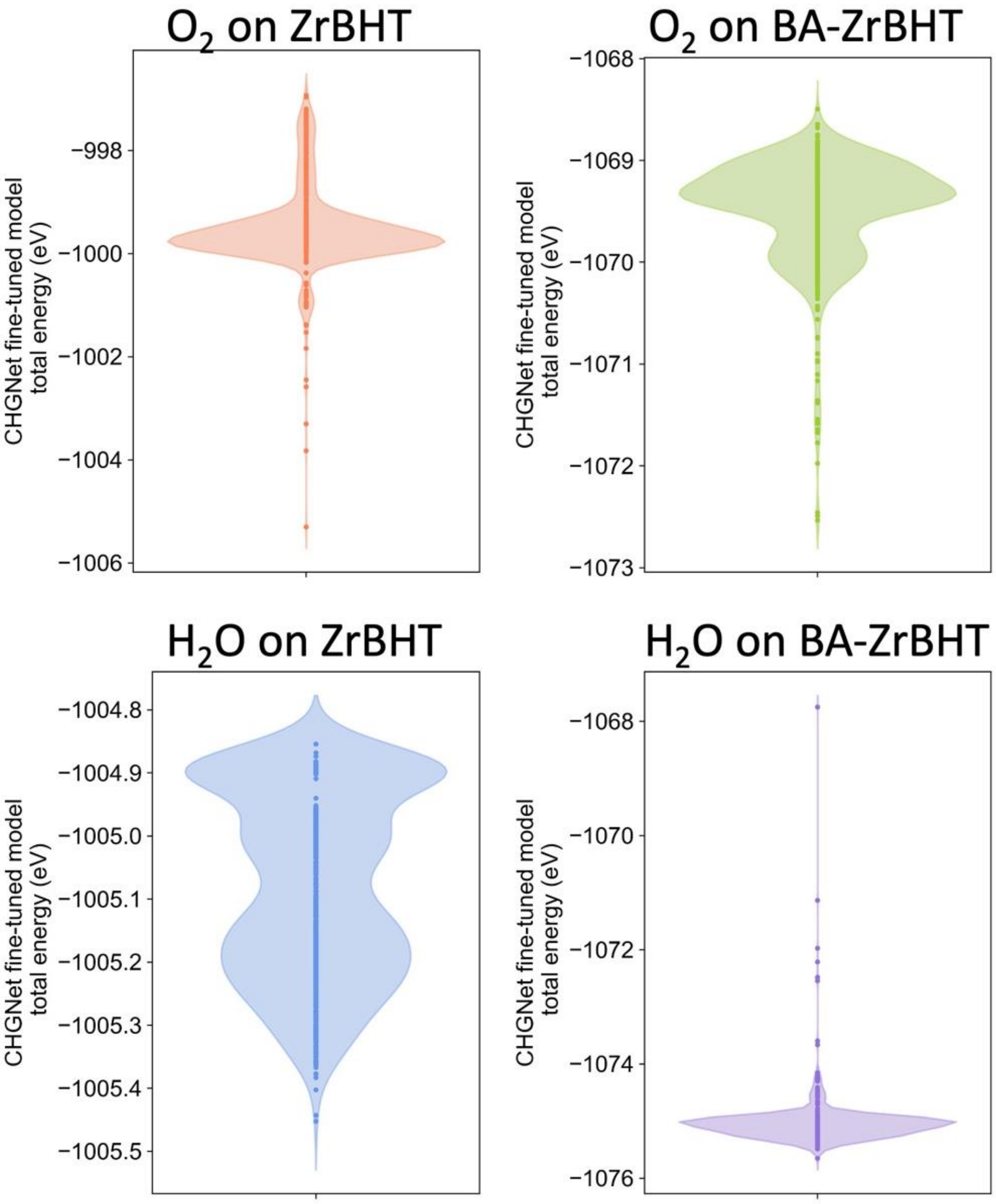

Figure S6. The calculated total energy via the fine-tuned CHGNet machine-learning model. The width of the violin plots indicates the relative number of data points.

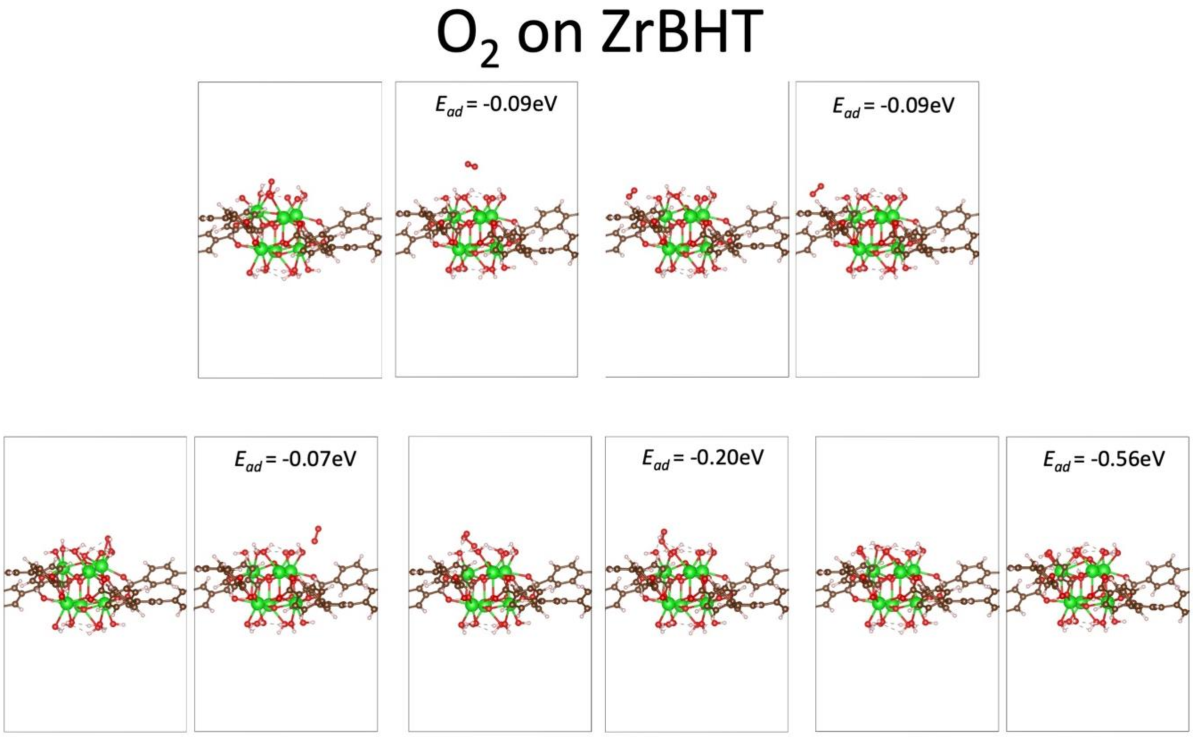

Figure S7. Structures optimized via DFT of various initial O_2 orientations on ZrBHT surfaces, alongside their corresponding adsorption energies (E_{ad}). The structures labeled with E_{ad} values represent the configurations after geometry optimization, while the structure to the left illustrates the orientation prior to optimization.

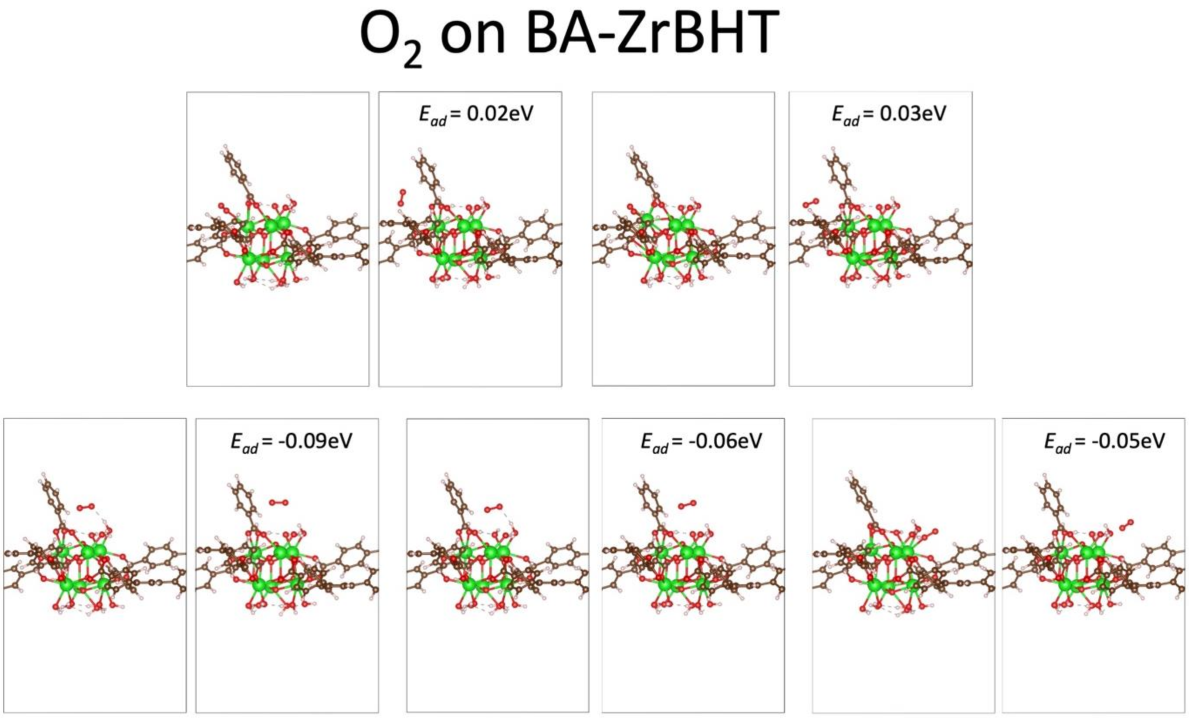

Figure S8. Structures optimized via DFT of various initial O_2 orientations on BA-ZrBHT surfaces, alongside their corresponding adsorption energies (E_{ad}). The structures labeled with E_{ad} values represent the configurations after geometry optimization, while the structure to the left illustrates the orientation prior to optimization.

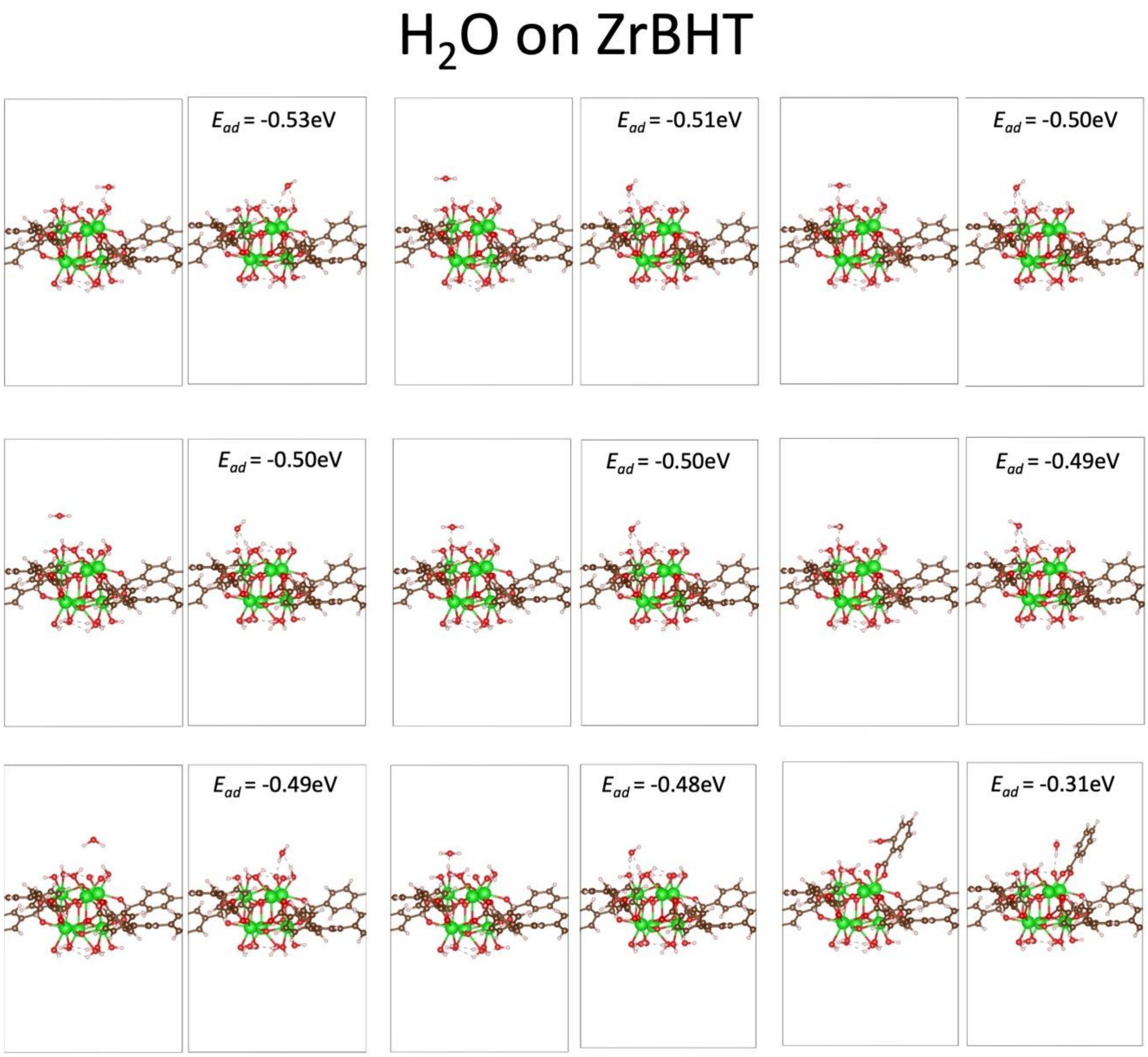

Figure S9. Structures optimized via DFT of various initial H_2O orientations on ZrBHT surfaces, alongside their corresponding adsorption energies (E_{ad}). The structures labeled with E_{ad} values represent the configurations after geometry optimization, while the structure to the left illustrates the orientation prior to optimization.

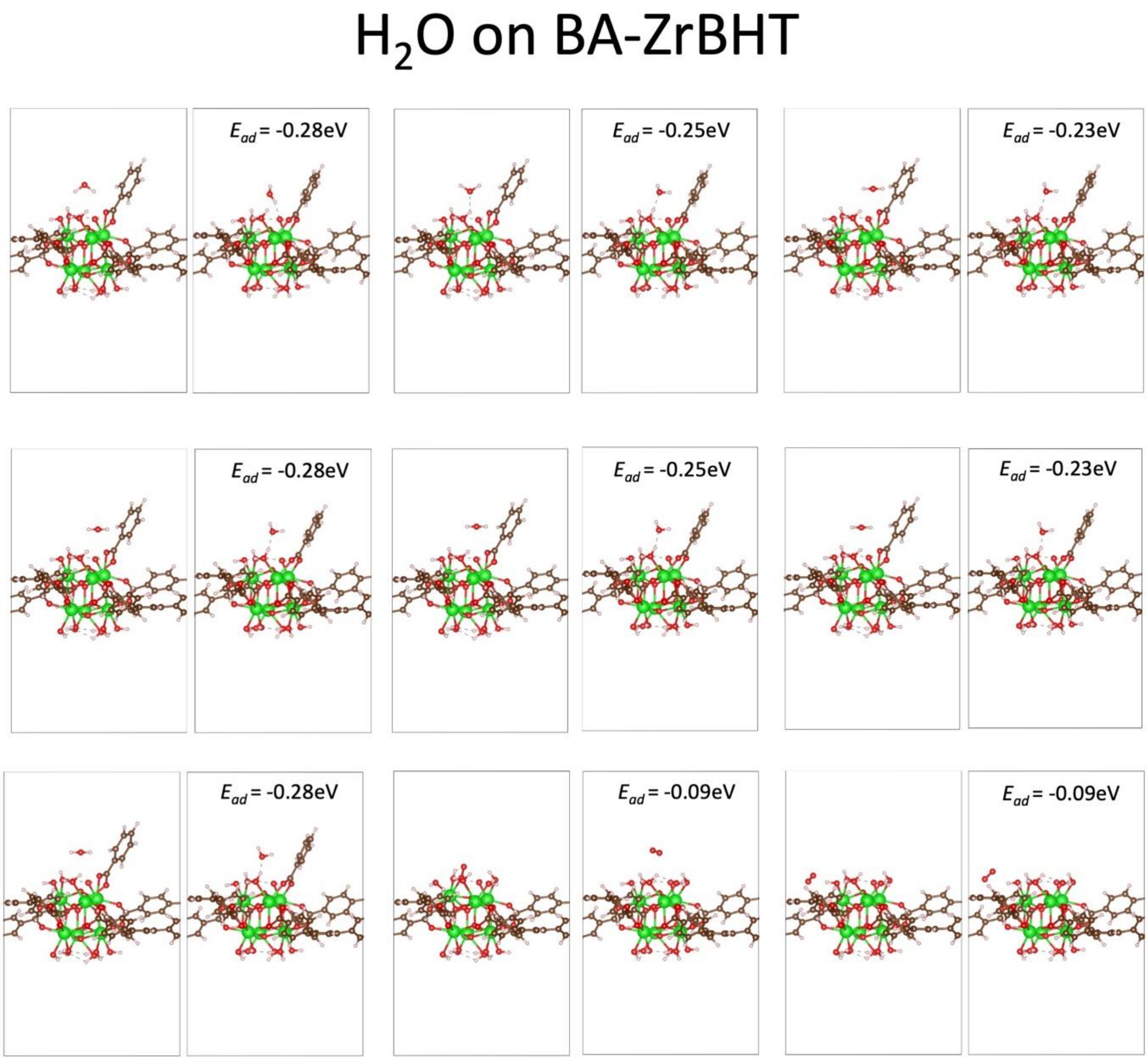

Figure S10. Structures optimized via DFT of various initial H_2O orientations on BA-ZrBHT surfaces, alongside their corresponding adsorption energies (E_{ad}). The structures labeled with E_{ad} values represent the configurations after geometry optimization, while the structure to the left illustrates the orientation prior to optimization.
